# Eliciting ERP Components for Morphosyntactic Agreement Mismatches in Perfectly Grammatical Sentences

**DOI:** 10.3389/fpsyg.2019.01152

**Published:** 2019-06-25

**Authors:** Émilie Courteau, Lisa Martignetti, Phaedra Royle, Karsten Steinhauer

**Affiliations:** ^1^Faculty of Medicine, School of Speech Language Pathology and Audiology, University of Montreal, Montreal, QC, Canada; ^2^Centre for Research on Brain, Language and Music (CRBLM), Montreal, QC, Canada; ^3^Faculty of Medicine, School of Communication Sciences and Disorders, McGill University, Montreal, QC, Canada

**Keywords:** subject-verb number agreement, event-related brain potentials (ERPs), auditory-visual sentence-picture matching paradigm, cross-modal number mismatches, French language, online grammaticality judgment, N400 and P600, sustained frontal negativity

## Abstract

The present event-related brain potential (ERP) study investigates mechanisms underlying the processing of morphosyntactic information during real-time auditory sentence comprehension in French. Employing an auditory-visual sentence-picture matching paradigm, we investigated two types of anomalies using entirely grammatical auditory stimuli: (i) semantic mismatches between visually presented actions and spoken verbs, and (ii) number mismatches between visually presented agents and corresponding morphosyntactic number markers in the spoken sentences (determiners, pronouns in liaison contexts, and verb-final “inflection”). We varied the type and amount of number cues available in each sentence using two manipulations. First, we manipulated the verb type, by using verbs whose number cue was audible through subject (clitic) pronoun liaison (liaison verbs) as well as verbs whose number cue was audible on the verb ending (consonant-final verbs). Second, we manipulated the pre-verbal context: each sentence was preceded either by a neutral context providing no number cue, or by a subject noun phrase containing a subject number cue on the determiner. Twenty-two French-speaking adults participated in the experiment. While sentence judgment accuracy was high, participants' ERP responses were modulated by the type of mismatch encountered. Lexico-semantic mismatches on the verb elicited the expected N400 and additional negativities. Determiner number mismatches elicited early anterior negativities, N400s and P600s. Verb number mismatches elicited biphasic N400-P600 patterns. However, pronoun + verb liaison mismatches yielded this pattern only in the plural, while consonant-final changes did so in the singular and the plural. Furthermore, an additional sustained frontal negativity was observed in two of the four verb mismatch conditions: plural liaison and singular consonant-final forms. This study highlights the different contributions of number cues in oral language processing and is the first to investigate whether auditory-visual mismatches can elicit errors reminiscent of outright grammatical errors. Our results emphasize that neurocognitive mechanisms underlying number agreement in French are modulated by the type of cue that is used to identify auditory-visual mismatches.

## Introduction

Few ERP studies have investigated real-time auditory sentence comprehension in French. Importantly, French subject-verb agreement has specific properties (such as clitic-verb liaison, see below) that are relevant to the study of agreement processing but have not received much attention in the ERP literature. Furthermore, many studies of agreement rely on visual word presentation, where morphosyntactic information is presented simultaneously with other lexical information, rather than unfolding over time, as in natural spoken language. These reading studies may not capture temporal aspects typical of spoken language processing, and ERP components may differ across modalities. Moreover, there is increasing interest in ERP methods that do not rely on violation paradigms. Considering these issues, we developed an ERP study where we implemented an auditory-visual sentence-picture matching task to investigate on-line processing of lexico-semantic and morphosyntactic information. Creating mismatches between grammatical auditory sentences and picture stimuli has been shown to elicit ERPs in lexico-semantic noun mismatches (e.g., Willems et al., 2008). To our knowledge, these mismatches between modalities have not been used to study morphosyntactic processing, nor lexico-semantic verb mismatches. Therefore, we examined whether the auditory presentation of a grammatical sentence combined with a picture that doesn't match its morphosyntactic features would elicit the same ERP components as in classic paradigms using ungrammatical sentences. Our innovative approach is motivated by the long-term aim of our research program, which is to study language processing in children with developmental language disorder (previously referred to as specific language impairment, SLI) using ecologically valid stimuli. Combining images and speech resembles other common activities such as shared picture-book reading, or watching documentary or educational videos, where an image is presented concurrently with an oral description. In these cases, people being read or spoken to might make predictions about what the reader will say, and notice any incongruencies, as participants were expected to do during our experiment. Thus, we investigate: (i) lexical-semantic mismatches between visually presented actions and spoken verbs, and (ii) auditory-visual subject number mismatches while varying number-cue types at different positions in the sentence. These manipulations should allow us to better understand how French-speakers handle semantic and grammatical cues online and should also elucidate if cross-modal paradigms elicit similar ERP components as classic within-sentence agreement violations. We will first review relevant ERP findings and then develop our research questions.

In ERPs, lexical-semantic processing is typically reflected by the centro-parietal N400 component between 300–500 ms after word onset (Kutas and Hillyard, 1980). This brain wave can be elicited by lexical-semantic expectancy violations (Steinhauer and Connolly, 2008; Kutas and Federmeier, 2011). Its amplitude may reflect processing effort during lexical retrieval (Lau et al., 2008) and post-lexical integration (Steinhauer et al., 2017), or it can be described as an error signal reflecting the difference between one's lexical-semantic expectations (i.e., the “current model”) and the actual word input (Bornkessel-Schlesewsky and Schlesewsky, 2019; henceforth BSS2019). Although most evidence for N400s has come from reading studies, this component has also been observed in bimodal (auditory-visual) lexical-semantic violations where an incongruous image is presented concurrently with an auditory utterance, for instance: *Je vois un* *!soulier* *vert sur la table* “I see a green !shoe on the table” with an image of a [HAT on a table] (Royle et al., 2013; see also Friedrich and Friederici, 2004; Willems et al., 2008). The N400 is generally considered a reliable ERP correlate of increased lexico-semantic processing difficulties.

Morphosyntactic agreement-error processing in reading studies is often indexed by one or two components, the left anterior negativity (LAN) and a later positive shift (the P600). The LAN has been reported for a range of morphosyntactic violations, including subject-verb agreement violations (e.g., *As a turtle grows, its shell* ^*^grow too Kutas and Hillyard, 1983), especially in languages with relatively free word order and rich morphological agreement marking (Angrilli et al., 2002; Barber and Carreiras, 2005), but also in languages with less rich paradigms (Osterhout and Mobley, 1995; Hagoort and Brown, 2000). Like the N400, this component typically emerges between 300 and 500 ms after stimulus presentation. Most agreement studies eliciting LANs have been conducted in the written modality, but some auditory studies have also reported LAN-like negativities for a range of morpho-syntactic anomalies (Friederici et al., 1993; Balconi and Pozzoli, 2005; Rossi et al., 2006; Hasting and Kotz, 2008; Morgan-Short et al., 2010; Dube et al., 2016; Haebig et al., 2017). Compared to reading studies, LANs in auditory studies tend to have an earlier onset, and can have a much longer duration (~100–1,200 ms), and a bilateral frontal distribution (e.g., Hasting and Kotz, 2008). However, several reading studies do not report LANs for agreement violations (Osterhout and Mobley, 1995; Tokowicz and MacWhinney, 2005; Lau et al., 2006; Nevins et al., 2007; Foucart and Frenck-Mestre, 2011, 2012) and report only P600s (see below). Whether or not LANs are reliable reflections of ERP morphosyntactic processes, whether different ERP morphologies reflect distinct processes, and what their functional significance may be, is therefore under debate (Molinaro et al., 2011a; Steinhauer and Drury, 2012; Royle et al., 2013; Tanner, 2015).

The LAN is usually followed by a late parietal positive-going component, the P600, roughly between 500 and 1,000 ms (Osterhout and Holcomb, 1992, 1993; Hahne and Friederici, 1999; Steinhauer et al., 1999). In contrast to the LAN, the P600 is widely viewed as the most consistent ERP signature for a large range of grammatical anomalies. It has been observed for morphosyntactic agreement violations (Frenck-Mestre et al., 2008; Foucart and Frenck-Mestre, 2011, 2012; Molinaro et al., 2011a; Royle et al., 2013), syntactic violations (Friederici, 2002), garden path sentences (Osterhout and Holcomb, 1992; Holcomb, 1993), and has also been elicited by semantic anomalies in conjunction with N400s (Hagoort, 2003; Steinhauer et al., 2010; Royle et al., 2013). While many agree that the P600 is a brain response related to controlled sentence reanalysis and repair (Hahne and Friederici, 1999), some argue that it is an ERP correlate of implicit syntactic processing (Tokowicz and MacWhinney, 2005). Another interpretation is that the P600 is a member of the parietal P300 (P3b) family of components reflecting stimulus categorization (e.g., in an acceptability judgment task) (Royle et al., 2013; Sassenhagen et al., 2014; BSS2019).

ERP studies have also revealed different patterns for various agreement error types. A majority of studies on agreement are reading tasks, and most use serial word-by-word visual presentation. Molinaro et al. (2011a) present a review of number and gender agreement processing in various languages. Regarding subject-verb number agreement violations, of 17 studies reviewed, all revealed P600s and 13 revealed LANs. The authors correlate the LAN with morphosyntactic error processing and explain the absence of a LAN in certain studies by differences in morphosyntactic saliency. For example, when these are underspecified (i.e., not morphologically expressed on the singular), a LAN may not be triggered. Molinaro et al. (2011b), found that in conditions such as ^*^Il ragazzo e la ragazza corre… “The boy and the girl run.3rd.singular,” the conjoined noun phrase (NP) does not contain any overt plural marking and in its absence no LAN is triggered. However, Frenck-Mestre et al. (2008) do not observe any negativities resembling a LAN but find a P600 in French native speakers in response to subject-verb agreement violations such as ^*^Le matin je mangez [mãʒe] “In the morning I eat.2nd.plural.” Their data contradict Molinaro's (2011a) interpretation, as the LAN was absent even though subject number properties were clearly expressed by the singular pronoun *je* “I” as well as the verb *mangez*.

In sum, while both the P600 and the LAN can be observed following various agreement-error types, it is still unclear whether they are modulated by the languages, structures, or contexts used to elicit them. The present study attempts to answer the following questions, using entirely grammatical sentences in all conditions. First, whether French speakers will elicit an N400 component for cross-modal (audio-visual) lexico-semantic mismatches realized on actions/verbs—rather than nouns/objects—and whether this violation type will elicit P600s as observed in other cross-modal lexico-semantic mismatch studies. Second, whether cross-modal number mismatches between the pictures' agents and the determiners/pronouns or verb morphology in our sentences elicit biphasic LAN/N400-P600 complexes as in previous morphosyntactic violation studies. To the best of our knowledge, this has not been investigated before. Given that our sentences were grammatical, one could argue that cross-modal number mismatches may cause either (a) conceptual-semantic problems typically associated with N400s instead of LANs, or (b) logical-semantic conflicts related to truth values, which have be found to elicit local N400s or sentence wrap-up effects (Bokhari, 2015) and P600s followed by (but not preceded by) late LANs (L-LANs; cf. Steinhauer et al., 2010). The third question was whether participants, when presented with multiple cues for number mismatch disambiguation, will rely on the first available auditory cue, as indicated by ERP responses.

## Materials and Methods

### Participants

Twenty-eight neurotypical adults aged 18–40 years participated in the experiment. The protocol was approved by Institutional Review Boards at McGill and University of Montreal (UdeM). All participants gave written informed consent in accordance with the Declaration of Helsinki. All were right-handed as assessed using the Edinburgh Handedness Inventory (Oldfield, 1971), had normal or corrected-to-normal vision, French as their mother tongue and their everyday language, and did not learn any other language before age 5. None had learning disabilities, neurological damage, or hearing loss. Working memory was assessed orally at session's end. Participants were recruited from Montreal university student populations. Participants were compensated $45 for their time (3.5 h). Six data sets had to be excluded due to excessive eye movement artifacts, such that data from 22 participants were retained for analyses (range: 18–38 years; mean 25; 12 female, 10 male). We consider this sample size as enough to provide a good estimate of the effects of interest, since in Royle et al. (2013) a group of 15 French-speaking adults participating in a similar paradigm (7 in a task-based group and 8 in a no-task one) showed significant ERPs related to adjective agreement errors and noun-image semantic incongruencies in each group.

### Materials and Design

As illustrated in Tables 1–3, materials consisted of spoken grammatical sentences in French, half of which mismatched with a concurrently-displayed picture, either through the action described or the number of agents (singular/plural mismatch). As we developed the study for younger populations (to be tested after adults), word selection was constrained by age-of-acquisition norms (see Supplementary Materials for details). Verbs were presented within sentences containing third person singular or plural subject pronouns (*he/she/they*), and a sentence continuation with a direct object NP, or prepositional phrase (PP, e.g., … *in the public pool*) to avoid sentence-final (or “wrap-up”) effects in ERPs time-locked to verbs (Hagoort, 2003; see also Stowe et al., 2018). Verbs were selected based on their number-agreement morphological characteristics, as explained below.

**Table 1:** Experimental sub-conditions for lexico-semantic manipulations and a corresponding visual stimulus.

**Visual stimulus**			
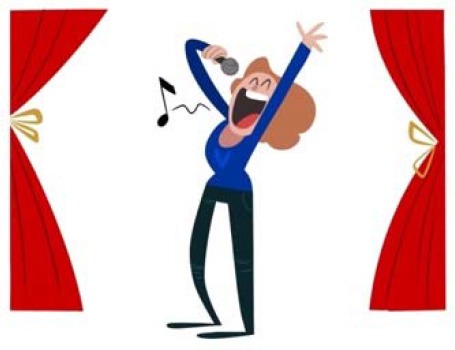	Sample visual stimulus presented concurrently with auditory stimuli for matching lexico-semantic conditions (1a-b) and mismatching ones (2a-b). Note that, in addition to the mismatch at the target verb (“sings” vs. “swims”), conditions 2a-b also include a second semantic mismatch in the prepositional phrase (here: “concert venue” vs. “public pool”).		
**Condition**	**Context**	**Sample**	**auditory stimuli**
Congruent semantics	Neutral	(1a)	*Chaque semaine | elle chante dans une salle de concert* “Each week | she sings at a concert venue”
	Subject NP	(1b)	*La vedette | elle chante dans une salle de concert* “The star | she sings at a concert venue”
Incongruent semantics	Neutral	(2a)	*Chaque semaine | elle !nage dans la piscine publique* “Each week | she !swims in the public pool”
	Subject NP	(2b)	*La vedette | elle !nage dans la piscine publique* “The star | she !swims in the public pool”

*Critical words are underlined. Subj NP, overt subject noun phrase; !, lexico-semantic mismatch; |, cross-splicing point*.

Selected critical verbs were inspired by the fLEX evaluation tool (Pourquie et al., 2016), with their imageability in mind, as they were presented alongside illustrations, and were matched on lemma frequency, age of emergence, and length (syllables and phonemes). Auditory stimulus recording, normalizing, and splicing was supervised by trained research assistants with a background in speech editing (Supplementary Materials). For each sentence, one color drawing was created by a professional artist, emphasizing the action being described, and the agent(s) carrying it out. Drawings maintained a constant visual complexity level, avoiding superfluous or distracting details.

In order to enhance the comparability of ERP effects between semantic and number mismatches, we decided to create semantic mismatches on the verb, the main element disambiguating mismatches in our number conditions (see below). Thus, for semantic mismatches, the spoken verb did not correspond to the depicted action (e.g., the sound file described “she swims…” and the image depicted “she sings…”). Sentences in this condition were created with 60 invariable regular verbs, 30 with a singular and 30 with a plural pronoun (“he/she,” “they”). Each pronoun+verb item was then combined with (a) a subject NP context providing a lexical NP with early number information (e.g., “*The*.*plural* *girls*, they swim”^1^ and (b) a neutral context without number information (e.g., “*In the evening*, they swim”), resulting in 120 spoken items. In total, 240 stimuli were created; 120 congruent and 120 in incongruent ones, by splicing the incongruent verb into the sentence (see e.g., Tables 1, 2A).

**Table 2:** Experimental sub-conditions involving liaison (LIAIS) verbs and corresponding visual stimuli.

**Visual stimulus**				
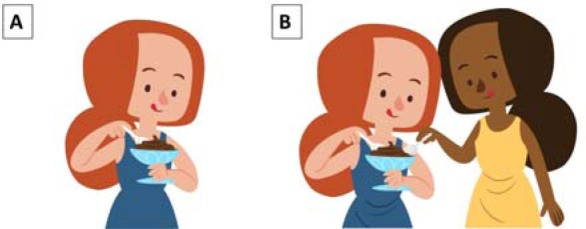		Image A: sample visual stimulus for match conditions (1a-b) and mismatch conditions (2c-d) in the singular. Image B: sample visual stimulus for match (2a-b) and mismatch conditions (1c-d).		
**Condition**	**Number**	**Context**	**Sample**	**auditory stimuli**
Congruent morphosyntax	Singular	Neutral	(1a)	*Au dessert | elle aime la mousse au chocolat* “For desert | she likes chocolate mousse”
		Subject NP	(1b)	*La fille | elle aime la mousse au chocolat* “*The* girl | she likes chocolate mousse”
	Plural	Neutral	(2a)	*Au dessert | elles⌣ aiment la mousse au chocolat* “For desert | they like chocolate mousse”
		Subject NP	(2b)	*Les filles | elles⌣ aiment la mousse au chocolat* “*The* girls | they like chocolate mousse”
Incongruent morphosyntax	Singular	Neutral	(1c)	Au dessert | elle ^*^ aime la mousse au chocolat “For desert | she ^*^likes chocolate mousse”
		Subject NP	(1d)	^*^La fille | elle ^*^ aime la mousse au chocolat “^*^The girl | she ^*^likes chocolate mousse”
	Plural	Neutral	(2c)	Au dessert | elles⌣^*^ aiment la mousse au chocolat “For desert | they ^*^like chocolate mousse”
		Subject NP	(2d)	^*^Les filles | elles⌣^*^ aiment la mousse au chocolat “^*^The girls | they ^*^like chocolate mousse”

Critical words are underlined. Subj NP, overt subject noun phrase;

* *, number mismatch; |, cross-splicing point*.

Number mismatches between the depicted subject and the one presented in the auditory stimulus (e.g., the sound file describes “she swims” and the image depicts “they swim”) were realized at different sentence positions using cross-splicing techniques (see Tables 2, 3).

**Table 3:** Experimental sub-conditions involving consonant-final (CONS) verbs, and corresponding visual stimuli.

**Visual stimulus**				
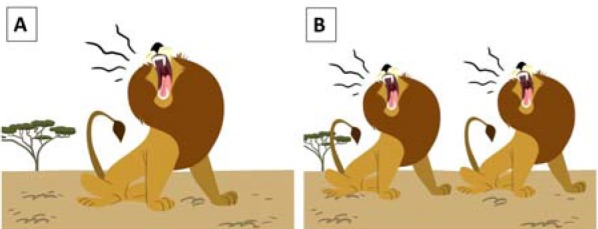		Image A: sample visual stimulus for match conditions (1a-b) and mismatch conditions (2c-d). Image B: sample visual stimulus for match (2a-b) and mismatch conditions (1c-d)		
**Condition**	**Number**	**Context**	**Sample**	**auditory stimuli**
Congruent morphosyntax	Singular	Neutral	(1a)	*En soirée | il *rugit* dans la savane* In the evening *|* he *roars* in the savannah
		Subject NP	(1b)	*Le lion | il rugit dans la savane* *The* lion *|* he *roars* in the savannah
	Plural	Neutral	(2a)	*En soirée | ils *rugissent* dans la savane* In the evening *|* they *roar* in the savannah
		Subject NP	(2a)	*Les lions | ils rugissent dans la savane* *The* lions *|* they *roar* in the savannah
Incongruent morphosyntax	Singular	Neutral	(1c)	En soirée | il ^*^rugit dans la savane During evening *|* he ^*^roars in the savannah
		Subject NP	(1d)	^*^Le lion | il ^*^rugit dans la savane The lion *|* he ^*^roars in the savannah
	Plural	Neutral	(2c)	En soirée | ils ^*^rugissent dans la savane In the evening *|* they ^*^roar in the savannah
		Subject NP	(2d)	^*^Les lions | ils ^*^rugissent dans la savane *The* lions | they ^*^roar in the savannah

Critical words are underlined. Subj NP, overt subject noun phrase;

* *, number mismatch; |, cross-splicing point*.

Two verb types were used: 60 liaison (LIAIS) verbs and 60 consonant-final (CONS) verbs. LIAIS verbs had vowel onsets and were regular 1st conjugation verbs, such as *aimer* “to-love,” which provide no audible cues or disambiguation between 3rd person singular (*aime* [εm]) and plural forms (*aiment* [εm]). This allowed us to ensure that the only cue for number disambiguation was located at the junction (liaison) between the subject pronoun and the verb, indexed by the presence or absence of the pronoun's plural marker “*s”* [z] (e.g., *elle aime* [εlεm] “she loves” vs. *elles*
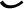
*aiment* [εl**z**εm] “they love”). Unlike LIAIS verbs, CONS verbs were from the 2nd and 3rd conjugation classes, such as *rugir* “to-roar,” where number distinctions between singular and plural forms are audible on verb endings (e.g., *il rugit* [ilʁyʒ**i**] “he roars” vs. *ils rugi****ss****ent* [ilʁyʒI**s**] “they roar”). This was the only number cue provided by CONS verbs. A total of 120 verbs (60 LIAIS and 60 CONS) were produced in singular and plural sentences, with both NP and neutral contexts. This resulted in 480 audio files and 960 stimuli: 480 in the congruent condition, and 480 in the incongruent one, where there was a mismatch between the spoken sentence and the picture's verb number.

The 1,200 different sentence-picture combinations (240 for conceptual semantics and 960 for agreement) were evenly distributed across four lists (with no sentence repetition within a given list). Three hundred stimuli sentences with accompanying images were presented to participants in each list (60 for conceptual semantics and 240 for morphosyntax) and were pseudo-randomized (see Supplementary Materials for details). Item versions for each condition were distributed across lists as follows: For semantics, one version of a given verb was included in each list, such that a participant heard one audio file and saw one image (either congruent or incongruent) for each verb. For each LIAIS and CONS verb type two sentence versions of a given verb were included in each list. These sentences were maximally distinct such that they differed in: (1) number (singular vs. plural), (2) context type (neutral vs. subject NP), and (3) congruency (match vs. mismatch with the image), and were presented in different halves of the experiment. This entailed that each subject be presented the same image twice (one match and one mismatch context), but with two completely different audio files.

### Procedure

Experimental sessions took place in a quiet room at the UdeM in the third author's lab. Upon arrival, participants read and signed the consent form, after which they completed the Edinburgh Handedness Inventory (Oldfield, 1971) and a language background questionnaire. They were then fitted with an EEG cap, and completed three sub-experiments, all of which used an auditory-visual sentence-picture matching paradigm. The first and second study examined gender-agreement processing (Royle et al., 2013) and word order in French noun phrases. Data from the third experiment are reported here. Total session duration was ~3.5 h, including consent form and other questionnaire completion, WM test administration, preparation, and clean up.

Participants were seated at a desk at a distance of ~40 cm from a computer monitor. Sentences and images were presented using an “Alien learning paradigm,” where an alien visited Quebec and was learning French. A story containing filler sentences, images and animations was created. These were interspersed throughout the experiment to maintain interest and attention. Participants listened to spoken sentences presented binaurally via insert earphones (ER-1 Insert Earphones, Etymotic Research), while images were presented on the computer monitor. A pause was programmed after every three experimental blocks (60 items).

Participants were instructed to listen to each sentence, while attending to all aspects of grammar and meaning, and judge sentence acceptability in relation to the simultaneously presented image by pressing one of two keys on a response pad (“acceptable” or “not acceptable”). In order to avoid laterality effects, the “acceptable” button was programmed on the right side of the pad for half the participants, and the left side for the other half. Participants were instructed to minimize movement and to keep their eyes open during stimuli presentation. Six practice trials were presented at experiment onset and were excluded from subsequent analyses. At least one researcher or assistant was present throughout the session. EEG recording was monitored throughout, and participants were given feedback about eye blinks and other body movements whenever necessary, in order to reduce artifacts.

Each trial began with a fixation cross centered on the screen 1,000 ms before stimulus presentation. The image was presented 500 ms before sentence onset, and stayed on screen until the auditory stimulus ended. Then, a response prompt (“???”) appeared on the screen until a response button was pressed, followed by a blank screen for 1,000 ms, during which subjects were instructed to blink their eyes before the next trial began.

### Analysis Time-Locking

**Figure 1:**
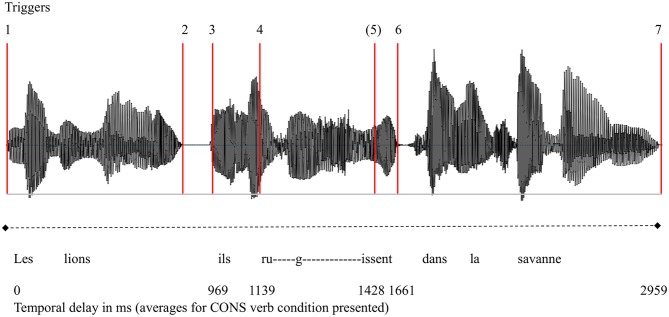
Example waveform of an auditory stimulus for the sentence *Les lions, ils rugissent dans la savane*. The red lines represent the various cue points, called “triggers,” measured in the audio file. Trigger 1, sentence onset; Trigger 2, context phrase offset; Trigger 3, pronoun clitic onset; Trigger 4, verb onset; Trigger 5, onset of verb-final consonant (only Type 2 verbs); Trigger 6, verb offset; Trigger 7, sentence offset.

In order to quantify the time course of number mismatch and lexical-semantic effects, our analyses were time-locked to relevant lexical-semantic and morphophonological cues (Steinhauer and Drury, 2012), using triggers at relevant speech signal positions. Figure 1 depicts an example waveform for the sentence *Le lion, il rugit dans la savane* “The lion, he roars in the savannah” as well as its trigger points. Analyses presented in this paper use triggers 1 (sentence onset) and 4 (verb onset).

### EEG Recording and Data Analysis

The EEG was recorded continuously with a 500 Hz sampling rate from 64 cap-mounted electrodes (WaveGuard caps, ANT; Enschede, NL) placed according to the extended International 10/20 system. The electrodes used for recording covered frontal, central, parietal, temporal and occipital lobes (FP1, FP2, F3, F4, F7, F8, Fz, C3, C4, Cz, P3, P4, Pz, T3, T4, T5, T6, O1, O2, Oz). All impedances were maintained below 5 kΩ and were checked every 45 minutes throughout the experiment. The EEG was amplified using an ANT Neuro Eego^TM^ sports amplifier referenced to the CPz electrode. All subsequent EEG/ERP data processing steps and analyses were carried out using EEProbe software package (ANT; Enschede, The Netherlands) and statistical analyses were performed in R (R Studio Team, 2015), Boston, MA^2^ using the Easy analysis and factorial experiments visualization package (Lawrence, MA. 2011, R package version 4.4-0^3^).

Offline, raw data were re-referenced to linked mastoids and filtered using a Gaussian bandpass filter of 0.3 to 40 Hz. Trials contaminated with eye blinks or other artifacts were rejected using a 30 μV criterion. All uncontaminated trials were entered into the final analysis. Using a 600 ms pre-stimulus baseline interval, single-subject EEG waveforms per condition were averaged separately over 2,100 or 3,100 ms epochs (−600 to 1,500 or 2,500 ms), time-locked to the relevant critical word onset (underlined words in Tables 1–3 above) and entered into grand average ERPs. After artifact rejection, an average of 48/60 trials for semantic mismatches and 192/240 trials for number mismatches were analyzed per participant. Based on visual inspection and the previous literature, we identified representative time-windows for statistical analyses of lexical-semantic and number mismatches, during which ERP components were quantified as the mean EEG signal voltage (in μV).

In all analyses, we compare mismatch conditions to their corresponding match conditions presenting the exact same spoken sentence but with a different picture. For example, a number mismatch analysis for singular sentences compares singular spoken sentences with subject NPs, combined with a corresponding picture showing one agent (match condition) or with a similar picture showing two agents (mismatch condition). ERP analyses for midline electrodes and lateral electrodes were performed separately. At midline electrodes, global ANOVAs for the semantic condition included 2 factors: condition (2 levels: mismatch vs. match), and Electrode position (4 levels: Fz, Cz, Pz, and Oz). At lateral electrodes, the global ANOVA included four factors: condition (2 levels: mismatch vs. match), hemisphere (2 levels: right vs. left), anteriority (3 levels: anterior vs. central vs. posterior), and laterality (2 levels: lateral vs. medial). For the *number* mismatch conditions, two additional factors were included for both analyses: context (neutral vs. subject NP) and number (singular vs. plural). Greenhouse-Geisser corrections were applied in order to address potential violations of sphericity. In these cases, the original degrees of freedom and corrected probability levels are reported. A hierarchically-organized analysis of variance was pursued whereby only theoretically relevant interactions (i.e., condition effects and their interactions with scalp distribution effects) and attendant *post-hoc* analysis results are reported. Given that the ERP effects of interest are generally observed close to the midline rather than at more lateral recording sites, 12 representative electrodes are used to illustrate effects, while head maps for difference waves cover the whole scalp.

Arcsine transformed accuracy data from acceptability judgments were analyzed using repeated-measure ANOVAs, computed separately for semantic and number (mis-)match conditions. The global ANOVA for number mismatches included four factors with 2 levels each: condition, context, gender, and number.

## Results and Interim Discussions

Following a reviewer's suggestion, we first present behavioral data, followed by ERP results and discussion for lexico-semantic mismatches, and finally results and discussion for number mismatches.

### Behavioral Data Results

Accuracy for acceptability judgments on *lexical-semantic* conditions were nearly at ceiling for both match and mismatch sentences (see Table 4), and a global ANOVA indicated no Condition effect (*p* < 1). Global ANOVAs for *number* mismatches on LIAIS verbs revealed significant main effects of Condition [*F*_(1, 21)_ = 6.39, *p* = 0.0196] in favor of matches, and Number [*F*_(1, 21)_ = 5.67, *p* = 0.0269] in favor of the plural (Singular: Mean 93.6, *SD* = 0.045; Plural: Mean = 95.7, *SD* = 0.048), qualified by interactions for Condition × Number [*F*_(1, 21)_ = 8.97, *p* = 0.0069], Condition × Context [*F*_(1, 21)_ = 5.90, *p* = 0.0242], and Number × Context [*F*_(1, 21)_ = 9.60, *p* = 0.0054]. All these interactions are primarily driven by lower rejection rates for singular mismatches in neutral contexts (in bold, Table 4), where number disambiguation was realized by the lack of a plural marker at the liaison. See section ERPs for Number Mismatches on Verbs for further discussion. A global ANOVA for CONS verbs revealed that these differed significantly by Condition [*F*_(1, 21)_ = 4.52, *p* = 0.0455], but no other significant effects were found. Mismatches were responded to less accurately than matches.

**Table 4:** Accuracy means (and standard deviations) for audio-visually matching and mismatching trials in lexico-semantic and number conditions for both liaison and consonant-final inflection conditions.

**Conditions**	**Match**	**Mismatch**
Semantics	93.9 (0.060)	92.5 (0.070)
Number: Liaison verbs	96.3 (0.035)	93.0 (0.066)
Singular: NP context	96.1 (0.069)	94.2 (0.082)
Singular: Neutral context	97.6 (0.036)	**86.5 (0.105)**
Plural: NP context	94.4 (0.074)	94.7 (0.079)
Plural: Neutral context	97.2 (0.046)	96.8 (0.087)
Number: Consonant-final verbs	94.8 (0.039)	91.6 (0.077)

*Sub-conditions (for number and context) are listed only where statistical analyses indicated different patterns (i.e., for LIAS verbs)*.

### ERPs for Lexico-Semantic Mismatches

**Figure 2:**
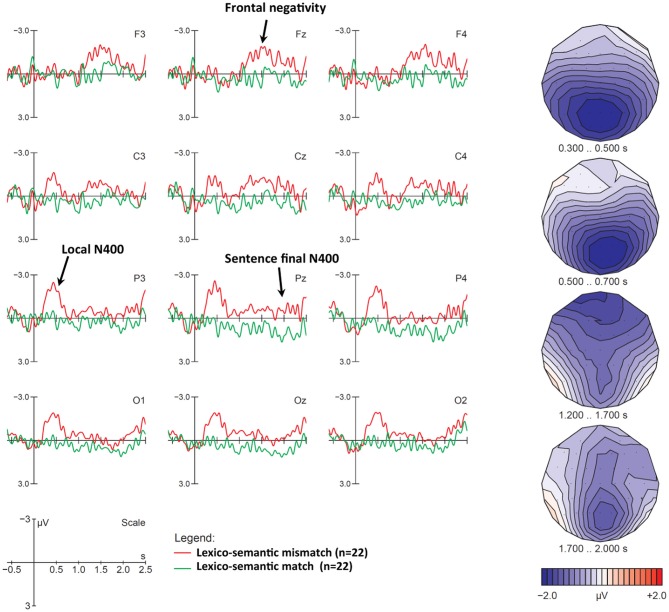
N400 and other negativities elicited by the lexico-semantic mismatch condition, collapsed across NP and neutral contexts. Displayed are grand-average ERPs at midline and eight lateral electrodes, as well as voltage maps illustrating the difference waves, for all participants, time-locked to the onset of the critical verb using a baseline of −600 to 0 ms. The vertical bar marks the onset of the critical verb. On average the verb ended 550 ms after onset; between 600 and 1,800 ms participants heard a NP/PP, which included a second semantic mismatch and ended the sentence. Compared with the correct match condition (green line), the semantic mismatches (red line) elicited a large extended N400 between 300 and 700 ms, followed by a frontal negativity during the NP or PP (1,200–1,700 ms), and a subsequent posterior sentence wrap-up N400 between 1,700–2,000 ms. Negative polarity is plotted upwards. Voltage maps represent difference waves (violation minus control), with negativities in blue and positivities in red. For illustration purposes only, ERP plots have been 10 Hz low-pass filtered.

As depicted in Figure 2, compared with the match condition, the semantic mismatch condition elicited a series of negativities across both context conditions at verb onset. First, we observe a posterior N400-like negativity between roughly 300–700 ms. Secondly a subsequent negative deflection emerges around 1,200 ms and lasts until 2,000 ms. It shows a frontal distribution until 1,700 ms and becomes more posterior afterward. Recall that the verb was always followed by an object noun phrase (NP) or a prepositional phrase (PP) that ended the sentence, and that nouns within these phrases also mismatched with the depicted information (see Table 1 for an example). On average, verbs ended 550 ms after onset, and participants heard the NP/PP between 600 and 1,800 ms. Based on this time course, we analyzed the negativities in five different time windows: 300–500 ms for the core N400, 500–700 ms for the extended N400, 700–1,100 ms for the interval that did not elicit effects, 1,200–1,700 ms for the negativity related to the NP/PP mismatch, and 1,700–2,000 ms for a presumed sentence-final N400-like negativity.

Statistical analyses for all time windows, separately for lateral and midline electrodes, are summarized in Table 5. Significant interactions in the global ANOVA were decomposed to identify scalp electrodes displaying the strongest condition differences. In both the 300–500 ms and 500–700 ms time windows, the most dominant and consistent effects included Condition × Anteriority interactions at both lateral and midline electrodes, as well as a Condition × Laterality interaction at lateral electrodes. Decomposing these interactions confirmed that the N400 reached significance only at posterior electrodes at or near the midline (Pz and Oz, and posterior medial electrodes). As expected, for the 700–1,100 ms time-window, we found no significant main effects or interactions involving Condition. As can be seen in Figure 2 (e.g., at Pz), the absence of an effect in this contrast cannot be attributed to the presence of a P600 that may have canceled out any ongoing negativities due to component overlap. In fact, there is not the slightest indication of a positive dip that could point to a “hidden” P600, including at posterior electrodes where P600s are usually found.

**Table 5:** Global repeated measures ANOVAs for lexico-semantic conditions (Trigger 4) at time-windows of interest.

			**(N400)**			**Late negativity**	**Wrap up effects**
		***df***	**300–500**	**500–700**	**700–1,100**	**1,200–1,700**	**1,700–2,000**
Lateral	Condition	(1, 21)	–	–	–	7.14^**^	–
	Condition × Anteriority	(2, 42)	5.26^***^	8.63^***^	–	6.28^**^	–
	Frontal: Condition	(1, 21)	–	–	–	9.82^**^	–
	Central: Condition	(1, 21)	–	–	–	5.02^*^	–
	Posterior: Condition	(1, 21)	8.08^**^	8.08^**^	–	–	–
	Condition × Laterarlity	(1, 21)	9.59^**^	5.34^*^	–	4.77^*^	–
	Medial: Condition	(1, 21)	5.44^*^	3.60^†^	–	7.06^*^	–
	Lateral: Condition	(1, 21)	–	–	–	5.26^*^	–
	Condition × Ant × Context	(2, 42)	–	5.26^*^	–	–	–
	NP context: Con × Ant	(2, 42)	–	13.16^***^	–	–	–
	NP context Ant: Con	(2, 42)	–	8.09^**^	–	–	–
	Condition × Lat × Ant	(2, 42)	–	–	–	4.51^*^	–
	Central: Con × Lat	(1, 21)	–	–	–	4.92^*^	–
	Central, medial: Con	(1, 21)	–	–	–	5.67^*^	–
	Posterior: Con × Lat	(1, 21)	–	–	–	10.13^**^	–
	Lateral: Con × Ant	(2, 42)	–	–	–	11.38^***^	–
	Lateral, frontal: Con	(1, 21)	–	–	–	11.21^***^	–
	Condition × Lat × Cont	(2, 42)	–	–	–	5.69^*^	–
	Neutral: Condition × Lat	(1, 21)	–	–	–	7.80^**^	–
	Neutral, medial: Con	(1, 21)	–	–	–	8.55^**^	–
Midline	Condition	(1, 21)	5.56^*^	–	–	10.26^***^	7.35^**^
	Condition × Electrode	(3, 63)	6.34^*^	10.79^***^	–	–	–
	Pz: Condition	(1, 21)	9.29^***^	7.35^**^	–	–	–
	Oz: Condition	(1, 21)	9.21^***^	12.85^***^	–	–	–

*Only significant results and trends are presented. Con, Condition; Ant, Anteriority; Lat, Laterality; Cont, Context*.

† p < 0.10,

* p < 0.05,

** p < 0.01, and

*** *p < 0.001*.

A global ANOVA for time-window 1,200–1,700 ms yielded a significant Condition effect at midline and lateral electrodes, as well as Condition × Anteriority, Condition × Laterality, Condition × Laterality × Anteriority, and Condition × Laterality × Context interactions. The first three interactions indicate that this broadly distributed late negativity is most prominent at frontal electrodes and along the entire midline, whereas it gradually decreases at more lateral and posterior sites over both hemispheres (see voltage map). Finally, decomposing the interaction involving Context, we found that the negativity was more broadly distributed in the NP context, but limited to medial electrodes in the neutral one. Global ANOVAs for the sentence “wrap-up” effect in the 1,700–2,000 ms time-window yielded a Condition main effect in the midline with no other interactions.

#### Discussion for N400 Effects

Lexico-semantic mismatches on verbs were reliably detected by participants and elicited a large N400 component, as expected. Importantly, our study focused on mismatches involving verbs/actions, and not nouns/objects as in Royle et al. (2013) and other previous studies. We have therefore demonstrated that an N400 can be reliably elicited in adult French native speakers in response to verb-action mismatches. We believe that these require more complex cognitive matching processes than noun-object pairings, as they involve syntactic and thematic relations between a verb and its arguments. For example, in order to appropriately illustrate the ditransitive verb *give*, one must include an agent, a patient, and a beneficiary.

After the classic N400 time-window (300–500 ms), the N400 continued until 700 ms post verb-onset. There are various possible interpretations for this finding. First, mismatches involving verbs rather than nouns may require more complex processing. Secondly, in auditory studies, the N400 sometimes shows a longer duration due to word variability across trials (Holcomb and Neville, 1990). Thirdly, extended N400s with durations up to 700 ms have been discussed as reflections of additional post-lexical integration. The relevant discussion concerns the N400's functional interpretation, and whether it simply reflects automatic expectancy-based processing (i.e., lexical access typically between 300 and 500 ms, Kutas et al., 2006; Federmeier, 2007; Lau et al., 2008) or whether it also reflects controlled post-lexical integration (i.e., spoken word integration into a higher-order meaning representation after 500 ms, e.g., Brown and Hagoort, 1993; Holcomb, 1993; Steinhauer et al., 2017). Fourthly, 2/3 of our verbs were immediately followed by a direct object, which, in this condition, also mismatched with the visual stimulus, and may therefore have elicited a second N400. Note that the negativity's scalp distribution between 500 and 700 ms resembled the N400 preceding it, such that it is impossible to rule out any of these explanations without additional analyses beyond the scope of this paper.

#### Discussion for Sustained Frontal and Posterior Negativities

Following N400 effects, we observed late sustained negativities, the first between 1,200 and 1,700 ms with a frontal distribution, and the second between 1,700 and 2,000 ms with a broad distribution, but a central-parietal maximum consistent with an N400. The frontal negativity was elicited while direct objects (NP) or prepositional phrases (PP) were being processed. Both the NP and the noun in the PP also mismatched with the picture (i.e., one sees a woman singing on a stage but hears “*she swims in the public pool*,” see Table 1). A comparison of this condition and the number mismatch conditions, where no incongruencies were present between the NP/PP in erroneous and correct sentences (see Figures 5 and 6 below), shows that we observe a sustained negativity between 1,200 and 1,700 ms only in the lexico-semantic mismatch condition, suggesting that it is related to this additional semantic mismatch. However, its frontal distribution is not typical of an N400 and may point to a combination of mismatch effects proper and frontal expectancy effects reflecting anticipation of an additional semantic mismatch. Similar effects have been found for anticipation of a predictable comma likely to render a sentence ungrammatical, and was interpreted as a contingent negative variation (CNV, Steinhauer, 2003). We interpret the late portion of the negativity as a potential “sentence wrap-up effect”, which we discuss in the section Sentence-Final Negativities and Wrap-Up Effects below.

#### Discussion for P600 Effects

Recall that the P600 has sometimes been elicited by semantic anomalies in conjunction with the N400, notably in a cross-modal mismatch paradigm (Royle et al., 2013), but also in purely auditory ones (Hagoort, 2003), and in reading studies (Steinhauer et al., 2010), and has therefore been argued to reflect mental monitoring and processing load related to language reanalysis (i.e., it is not specific to grammatical processing; Kolk et al., 2003; Steinhauer and Connolly, 2008; van de Meerendonk et al., 2009). Others have argued that these positivities are tightly linked to acceptability judgment tasks, potentially as a linguistic variant of the P300 component (Coulson et al., 1998; Friederici et al., 2001; Sassenhagen et al., 2014). The absence of positivities in the lexico-semantic condition, despite our use of a judgment task, may be explained by our particular mismatches. First, as reflected by the subsequent frontal negativities, participants seemed quite engaged in anticipating and processing additional semantic mismatches in the following NPs and PPs, and may not have categorized the sentence as unacceptable when encountering semantic mismatches on verbs. Another possibility is that semantic mismatches realized on verbs do in fact involve more complex conceptual-semantic processing than those realized on nouns and may draw attention away from whatever processes may elicit positivities found on nouns. As we are not aware of any other ERP studies using verb/action mismatches, this would need to be further investigated. Finally, P600s are certainly not a consistent finding for conceptual mismatches: the motivation for explaining their absence is primarily based on their presence in a recent study from our lab that used a very similar cross-modal paradigm (Royle et al., 2013). Perhaps the most important point is that the absence of a P600 in our semantic mismatch condition contrasts with the P600s observed in other mismatch conditions that we will discuss next.

### ERPs for Number Mismatches

#### Sentence Onset Effects

**Figure 3:**
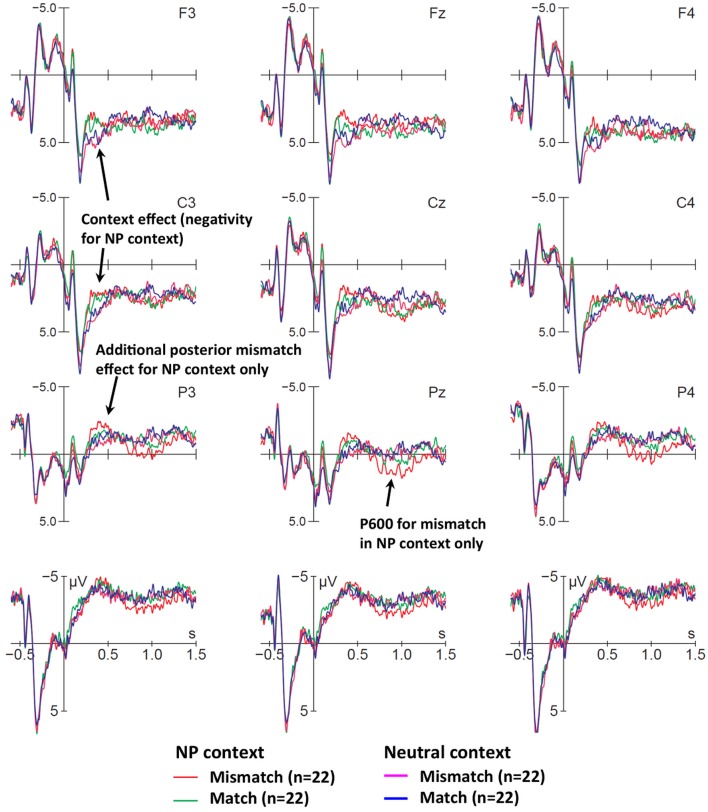
Early ERP effects of context and number mismatches at sentence onset. Displayed are grand-average ERPs at midline and lateral electrodes for all participants, time-locked to the onset of the determiner (vertical bar) with a baseline of −600 to 0 ms. Compared with neutral context correct (blue), and neutral context mismatch (magenta), the NP context correct condition (green), and the NP context mismatch condition (red) elicited an early negativity (300–500 ms). Furthermore, number mismatches with NP context display a small increased negativity (between 300–450 ms) and a large positivity between 700 and 1,200 ms. The two neutral conditions will be disambiguated further downstream at the verb and do not yet show differences at sentence onset.

At sentence onset we observed distinct ERP patterns for neutral contexts (with no disambiguation at this point) and NP contexts, where the NP either matched or not with the picture in number at the determiner (*le/la/les* “the.m.sg/f.sg/pl”). The distinction between LIAIS and CONS verbs does not play a role at this point, such that we can collapse across these conditions, which we did. Figure 3 displays match and mismatch conditions for both NP and neutral contexts, collapsed across singular and plural sub-conditions. Recall that the mismatch in neutral contexts happens only downstream on the verb and is, therefore, not yet expected to elicit mismatch components. The first 900 ms (−600 to 300 ms) are largely dominated by visual onset components (most prominently at occipital electrodes) for pictures (presented at −500 ms) and by auditory onset components (most prominently at fronto-central electrodes) for spoken sentences (starting at 0 ms), respectively. As can be seen, all conditions are virtually indistinguishable up to 300 ms after sentence onset, at which point the first context-effect emerges.

NP contexts, compared to neutral ones, elicited an early slightly left-lateralized fronto-central negativity (300–450 ms) after determiner onset. In the same time-window, we observe an additional enhanced negativity for NP context mismatches, which is followed by a P600 (700–1,200 ms). We will show how singular and plural mismatches in NP contexts contribute to this pattern. In neutral context conditions as expected no clear differences are visible, as confirmed by the absence of significant effects in all time-windows discussed below (see also Table 6). We return to neutral contexts at later sentence positions—at verb onset—where they are disambiguated.

**Table 6:** Global repeated measures ANOVAs for sentence onset effects (Trigger 1) at time-windows of interest.

			**(N400)**	**(P600)**
		***df***	**300–450**	**700–1,200**
Lateral	Condition	(1, 21)	–	–
	Context	(1, 21)	29.03^***^	2.99^†^
	Condition × Context	(1, 21)	5.32^*^	–
	Condition × Lat × Cont	(1, 21)	6.70^*^	–
	Condition × Ant × Cont	(2, 42)	–	7.95^**^
	NP context: Ant × Cond	(2, 42)	–	9.56^***^
	NP context, Post: Cond	(1, 21)	–	11.69^***^
NP context	Condition × Anteriority	(2, 42)	–	7.89^**^
	Posterior: Condition	(1, 21)	–	10.95^*^
	Condition × Ant × Hem × Num	(2, 42)	–	4.56^*^
	Posterior: Condition × Hem × Num	(1, 21)	–	10.95^*^
	Con × Ant × Hem × Num × Lat	(2, 42)	–	5.81^*^
	Left Hem: Condition × Ant	(2, 42)	–	7.80^**^
	Left Hem: Condition × Ant × Num	(2, 42)	–	5.97^*^
	Left Hem Sg: Condition × Ant	(2, 42)		10.49^***^
	Left Hem Sg Front: Condition	(1, 21)	–	3.23^†^
	Left Hem Sg Post: Condition	(1, 21)	–	5.03^*^
	Left Hem Pl: Condition × Ant	(2, 42)		3.90^*^
	Left Hem: Condition × Ant × Num × Lat	(2, 42)	–	4.25^*^
Midline	Condition	(1, 21)	–	–
	Context	(1, 21)	20.56^***^	9.58^**^
	Condition × Context	(1, 21)	9.78^**^	–
	NP context: Condition	(1, 21)	4.43^*^	–
	Condition × Elec × Context	(3, 63)	–	8.52^***^
	Pz: Condition	(1, 21)	–	8.44^**^
	Pz: Condition × Context	(1, 21)	–	6.22^*^
	Pz, NP: Condition	(1, 21)	–	12.10^***^
	Oz: Condition × Context	(1, 21)	–	10.92^*^
	Oz, NP: Condition	(1, 21)	–	9.34^**^
NP context	Condition	(1, 21)	4.43^*^	9.14^***^
	Pz: Condition	(1, 21)	–	11.35^***^
	Oz: Condition	(1, 21)	–	8.36^**^

*Only significant results and trends are presented. Ant, Anteriority; Con, Condition; Cont, Context; Elec, Electrode; Front, Frontal; Hem, Hemisphere; Lat, Laterality; Num, Number; Pl, Plural; Post, Posterior; Sg, Singular*.

† p < 0.10,

* p < 0.05,

** p < 0.01, and

*** *p < 0.001*.

##### ERPs for singular and plural mismatches in NP contexts

**Figure 4:**
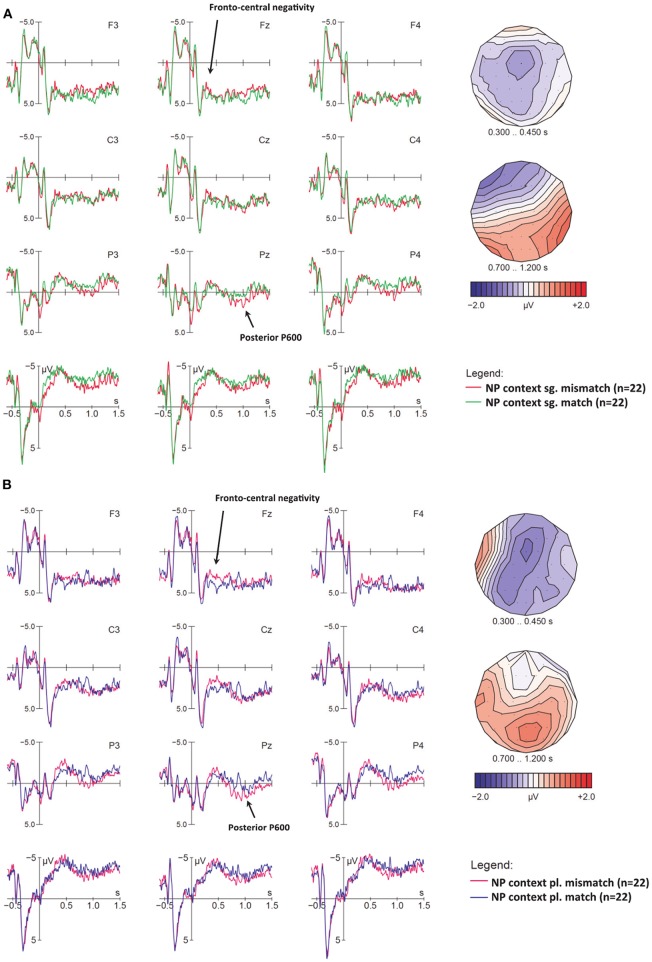
Early effects of cross-modal number mismatches in NP contexts, for **(A)** singular and **(B)** plural NPs at sentence onset. ERPs are time-locked to the onset of the determiner (vertical bar) with a baseline of −600 to 0 ms; voltage maps illustrate the difference waves of relevant effects. **(A)** Singular mismatches (red) elicited a small fronto-central negativity in the N400 time-window relative to singular matches (green), as well as a parietal P600. **(B)** Plural mismatches (magenta) elicited a larger N400 as well as a parietal P600, as compared to plural matches (blue). Voltage maps of these effects (mismatch minus control) show that singular and plural mismatches elicited quite similar components.

For sentences with *singular* NPs, we observe a small fronto-central negativity in the N400 time-window, followed by a posterior P600 in the mismatch condition between 700 and 1,200 ms after sentence onset (see Figure 4A). In the *plural* contrast (Figure 4B), we see a similar biphasic pattern for mismatches. However, the fronto-central negativity appears slightly larger and seems to extend more clearly to left posterior electrodes.

Statistical analyses for sentence-initial positions are summarized in Table 6. Global ANOVAs in the 300–450 ms time-window yielded a highly significant Context main effect. Mismatch effects were reflected by Condition × Context interactions in midline and lateral electrodes, as well as a Condition × Context × Laterality interaction in lateral electrodes. These interactions confirmed that the negativity for visuo-auditory number mismatches was limited to disambiguating NP contexts, and was largely limited to medial electrodes. Surprisingly, the absence of significant Anteriority and Number interactions suggested that (a) the apparent frontal focus of the negativity was not reliable across subjects and (b) the apparent differences in size and scalp distribution of negativities between singular and plural conditions (Figure 4A vs. Figure 4B) were not meaningful. Statistically, there was only a broadly distributed negativity in both singular and plural mismatches with NP contexts.

In the P600 time window (700–1,200 ms), global ANOVAs yielded a significant Condition × Electrode × Context interaction at midline electrodes, and Condition × Context and Condition × Laterality × Context interactions in lateral electrodes (see Table 6). Decomposing these interactions confirmed that the P600 had a posterior distribution and was limited to number mismatches in NP contexts. While this P600 was consistent across singular and plural at midline electrodes (significant Condition main effect at Pz and Oz), additional interactions with factor Number and topographical factors at lateral electrodes indicated that only for singular mismatches the P600 time-window also showed a (non-significant) frontal negativity over the left hemisphere. Overall, both singular and plural mismatches with NP contexts elicited consistent P600s that lasted until 1,200 ms.

Note that this relatively long P600 duration means that this effect was still present when the verb was presented (average verb onset at 1,140 ms, *SD* = 149 ms) and would have contaminated baselines and ERP analyses time-locked to verb onset (see Steinhauer and Drury, 2012). For these reasons, we refrained from analyzing the NP-context conditions at the verb, even though it would have been interesting to see whether additional disambiguating information elicited more mismatch effects further downstream.

##### Discussion for sentence-initials effects

Independent of mismatches, context manipulations at sentence onset elicited a larger negativity for NP contexts between 300 and 450 ms after sentence onset: this was likely triggered by the first word. Both NP contexts and neutral contexts started with function words (e.g., *Au dessert* “at-the desert” = “for desert” in neutral contexts, *La/les fille/s* “The girl/s” in NP contexts) for which N400 effects are rather atypical. In addition, the context-driven negativity had a more frontal distribution than a classic N400. We speculate that this context main effect may reflect enhanced alertness once participants had identified that a sentence started with a determiner and could, therefore, provide the first disambiguating task-relevant cue.

Interestingly, determiner *mismatches* elicited an additional, more broadly distributed negativity in virtually the same time-window, which was followed by a posterior P600, for both singular and plural mismatches. The mismatch negativity could be interpreted either as a lexical prediction effect (i.e., an N400, Tanner and Van Hell, 2014; BSS2019) or an effect of reference resolution (i.e., an N-ref component, e.g., Van Berkum et al., 1999). In the first scenario, participants would expect a specific determiner coherent with the number (and gender) of depicted potential subjects, and process a mismatch as a lexical (or phonological) error. In the second scenario, participants might wonder, when there are multiple potential subjects, who *la fille* ‘the girl' refers to. However, reference resolution effects only seem to make sense—and have only been reported—for singular nouns where contexts provide multiple potential referents, while we found no statistical differences between our singular and plural conditions and, moreover, we found them at the determiner rather than the noun. For these reasons we believe that this negativity reflects a mismatch for specific predictions. Our finding is reminiscent of that by DeLong et al. (2005) who reported an N400 on determiners for unexpected sentence continuations after a highly constraining context (e.g., *an airplane* rather than *a kite* after “… the boy went outside to fly_”). Whether this effect is primarily lexical or phonological in nature remains unclear.

The following P600-like positivity in our data may either reflect (a) an immediate categorization of the sentence as unacceptable (Sassenhagen et al., 2014) or (b) cross-modal integration of conflicting number information as in previous morphosyntactic (dis-)agreement studies, possibly linked to structural disambiguation or revisions (see e.g., Molinaro et al., 2011a, for a review), or both. In line with our previous work and the literature (e.g., Friederici et al., 2001; Steinhauer and Connolly, 2008; Royle et al., 2013), we maintain the view that the P600 typically reflects multiple cognitive processes and comprises multiple subcomponents. A P600 account involving structural (rather than purely lexical) mismatches or revisions would imply that participants in our study syntactically integrated the determiner with the subsequent noun, which was phonologically compatible with both a singular and a plural form (*fille/s* [fij]). However, a picture of two girls would have suggested (and pre-activated) a plural referent, which then mismatched with the spoken singular determiner (*la* “the.sing.fem”), thereby resulting in a traditional number agreement violation (i.e., *la* ^*^filles). Given that these early-disambiguating contexts were followed by additional information disambiguating subject number on the verb, one might expect higher confidence (and thus higher accuracy) in grammaticality ratings compared to sentences with neutral contexts. However, as discussed above (see also Table 4), this was not the case, supporting immediate categorization at the first available cue. We anticipate that this pattern may be different in children, especially those with language impairment, who are currently being tested with this same paradigm.

For obvious reasons, number mismatch effects at sentence-*initial* words (as in our study) are absent from the previous literature as they can only be created in relation to a previously presented context (here: a picture). Overall, it is remarkable that this sentence-initial number mismatch elicited an N400-P600 pattern previously found for morpho-syntactic agreement violations. It suggests that non-linguistic visual information from the environment can be immediately used (in < 500 ms) to make strong predictions about appropriate linguistic representations, or that “feature checking” processes are not constrained to linguistic representations. The elicitation of a P600 at this early position in a sentence is clearly compatible with accounts of “conflict monitoring” (Kolk et al., 2003) and “well-formedness categorization” (Sassenhagen et al., 2014), but more difficult to explain in terms of a structural “reanalysis” (Friederici, 2002).

#### ERPs for Number Mismatches on Verbs

We will now turn to mismatch effects at target verbs in neutral contexts. At sentence onset, LIAIS and CONS verbs did not differ, but at trigger 4 (verb onset) they did, because for LIAIS verbs, number disambiguation is available at verb onset (e.g., *elles[z]aiment* “they like”), while for CONS verbs, this information is available only at the verb final phoneme (e.g., *ils rugissent* [*ʁy*ʒI**s**] “they roar”). We will first focus on LIAIS verbs and then turn to CONS ones and consider only neutral contexts because these are the ones being disambiguated for the first time on the verb.

##### ERPs for liaison verbs at verb onset at Trigger 4

**Figure 5:**
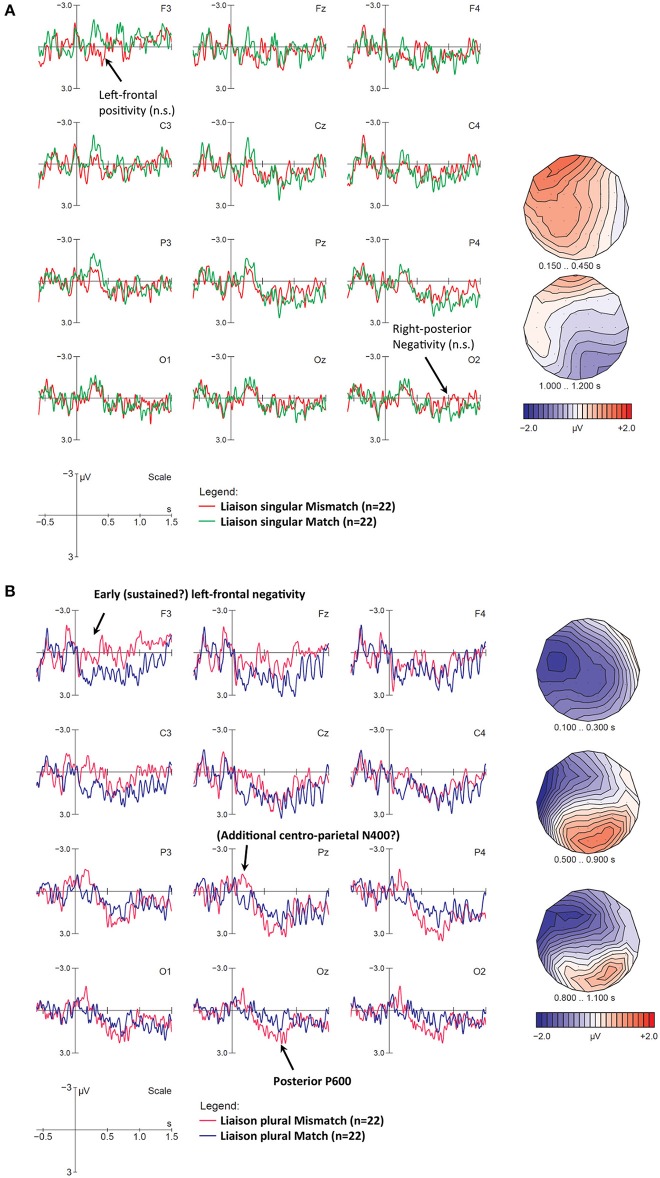
ERP effects for number mismatches at liaison verbs with neutral context, **(A)** for singular and **(B)** for plural verbs. Displayed are grand-average ERPs at midline and lateral electrodes for all participants, time-locked to the onset of the liaison using a baseline of −600 to 0 ms. The vertical bar marks the onset of the liaison. **(A)** For singular verbs, neither the early frontal positivity between 150 and 450 ms nor the posterior negativity (1,000–1,200 ms) reached significance. **(B)** Compared to the correct control condition (blue lines), plural mismatches (magenta lines) show early negativities (100–300 ms), followed by a posterior P600 (500–900 ms). After the end of the P600, a negativity seems to re-emerge at frontal and central electrodes (third voltage map).

As with sentence initial effects, we analyzed singular and plural violations separately. Figure 5A shows number mismatches time-locked to singular LIAIS verbs. In this comparison we did not observe the expected pattern but rather an apparent early left-anterior positivity between 150 and 450 ms after verb onset, and a posterior right-lateralized late negativity between 1,000 and 1,200 ms. However, global ANOVAs on singular LIAIS verbs in neutral conditions yielded no significant effects involving Condition at either the midline or lateral electrodes. (Note that the very early left-frontal positivity was partly driven by one participant's enhanced horizontal eye movements in this condition only, resulting in a polarity inversion of this difference between left-anterior and right-anterior electrodes—especially F7 and F8. Analyses excluding this data set did not change results, however. For consistency, we decided to present ERP data including this data set). Overall, our analyses did not point to any consistent ERP pattern for these number mismatches. Recall that this was also the condition with the lowest overall accuracy rate in our mismatch conditions (Table 4).

As illustrated in Figure 5B, for plural mismatches we observed an early left-lateralized fronto-central negativity between 100 and 300 ms, followed by a posterior P600-like positivity (500–900 ms), which then seems to be followed by a second late frontal and somewhat left-lateralized negativity from ~800 to 1,200 ms. In fact, when inspecting the left-anterior electrode F3 alone, the patterns looks like a sustained early negativity, starting around 100 ms and lasting until ~1,400 ms.

ANOVAs for plural verbs in the 100–300 ms time window yielded a significant Condition main effect at midline and lateral electrodes, and a Condition × Laterality interaction in lateral electrodes (see Table 7). This interaction means that the negativity was strong at medial electrodes, but only marginally significant at more lateral electrodes. Given that the early negativity seemed most prominent over left-frontal electrodes (especially F3), the lack of interactions involving factors Hemisphere or Anteriority was somewhat surprising. However, this was due to the fact that (a) the negativity was stronger at medial than lateral electrodes over *both* hemispheres, and (b) at posterior electrodes, the negativity was almost equally strong over both hemispheres (suggesting a second and more posterior N400-like negativity near the midline). An ANOVA in the P600 time-window (500–900 ms) yielded significant interactions of Condition × Electrode at midline, and Condition × Anteriority as well as Condition × Hemisphere at lateral electrodes. These interactions point to a posterior P600 co-occurring with an ongoing left-frontal negativity that gains strength once the P600 dissipates. In fact, between 800 and 1,100 ms we found a significant Condition effect at F3 (*p* < 0.02) and Fz (*p* < 0.03), but not at more posterior electrodes. This pattern of an early frontal negativity and its reoccurrence after an intervening positivity is reminiscent of that previously described for various syntactic violations in auditory ERP studies (Steinhauer and Drury, 2012), suggesting a sustained frontal negativity and a temporarily overlapping P600. We will return to this below.

**Table 7:** Global repeated measures ANOVAs for liaison verbs (LIAIS) in neutral contexts, for both singular and plural (Trigger 4) at time-windows of interest.

			**(LAN)**	**(P600)**
		***df***	**100–300**	**500–900**
**NEUTRAL CONTEXTS SINGULAR VERBS**				
Lateral	Condition	(2, 42)	–	–
	Condition × Anteriority	(2, 42)	–	–
Midline	Condition	(3, 36)	–	–
	Condition × Electrode	(3, 36)	–	–
**NEUTRAL CONTEXTS PLURAL VERBS**				
Lateral	Condition	(1, 21)	6.39^*^	–
	Condition × Laterality	(1, 21)	6.12^*^	–
	Medial: Condition	(1, 21)	7.22^**^	–
	Lateral: Condition	(1, 21)	3.63^†^	–
	Condition × Anteriority	(2, 42)	–	6.66^**^
	Condition × Hemisphere	(1, 21)	–	4.40^*^
Midline	Condition	(1, 21)	6.20^*^	–
	Condition × Electrode	(3, 36)	–	5.23^*^

*Only significant results and trends are presented*.

† p < 0.10,

* p < 0.05, and

** *p < 0.01*.

##### ERPs for consonant-final verb conditions at Trigger 4

**Figure 6:**
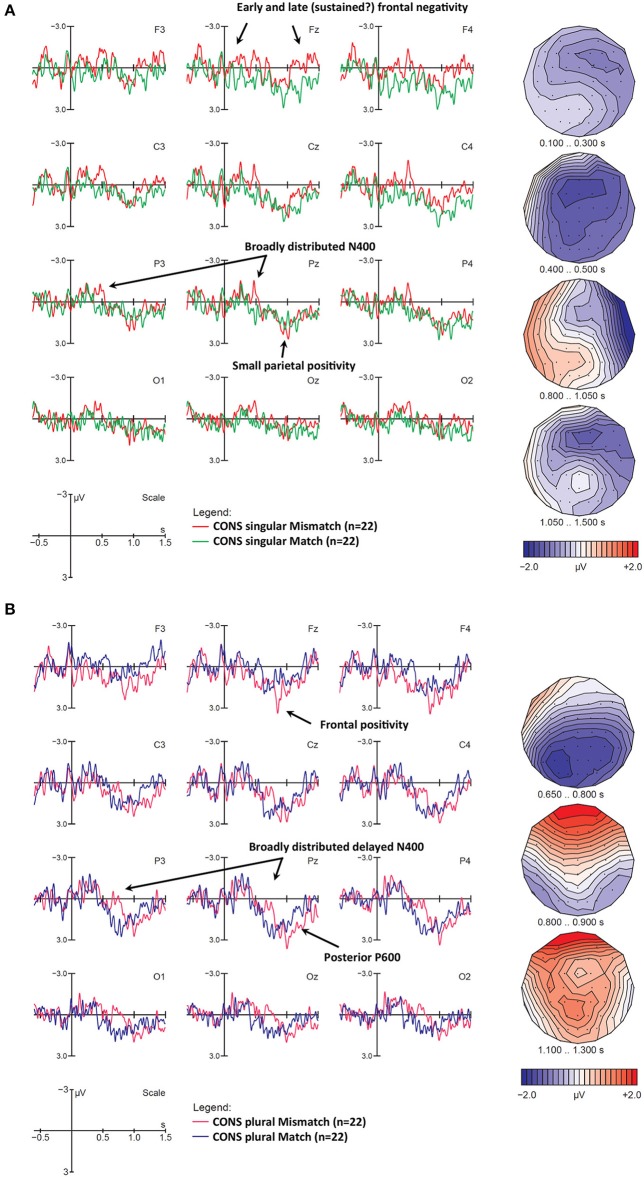
ERP number mismatch effects at consonant-final verbs with neutral contexts, **(A)** for singular and **(B)** for plural verbs. Displayed are grand-average ERPs at midline and lateral electrodes as well as voltage maps illustrating the difference waves, for all participants, time-locked to the onset of the critical verb using a baseline of −600 to 0 ms. The vertical bar marks the onset of the critical verb. **(A)** Compared to the match condition (green lines), singular mismatches (red lines) show an early sustained negativity at frontal electrodes (100–1,500 ms; cf. Voltage maps 1 and 4), an additional N400 (400–500 ms), and an intermediate time window during which a right-anterior negativity and a left-posterior negativity seem to cancel each other out along the midline (800–1,050 ms). **(B)** Compared to the match condition (blue lines), plural mismatches (magenta lines) show an N400-like negativity (650–800 ms), followed by a frontal positivity (800–900 ms) and a posterior P600 (1,100–1,300 ms).

While liaison verbs phonologically disambiguated number at verb onset, consonant verbs provided number information on the verb-final “morpheme” consonant. Due to this difference, one would expect mismatch effects to occur somewhat later than for liaison verbs. As shown in Figure 6A, for mismatch CONS *singular* verbs, the most prominent difference between match and mismatch conditions was a broadly distributed, slightly right-lateralized negativity in the N400 time window (400–500 ms after verb onset), which does not seem to be followed by a clear positivity in the P600 time-window. Note however that at anterior electrodes the N400 is both preceded and followed by a negativity starting around 100 ms, which seems to end around 600 ms and re-occur around 1,000 ms. This pattern could, once again, reflect temporary ERP-component overlap, namely an early but sustained negativity with a frontal maximum (from 100 to 1,500 ms), which is superimposed first by a parietal N400 that temporarily results in a more posterior scalp distribution (from 400 to 500 ms) and then by a left-lateralized and posterior positivity (from 800 to 1,000 ms) that temporarily cancels out the negativity at most electrodes (especially over the left hemisphere), until the frontal negativity re-emerges. The assumption that the early (100–300 ms) and late negativity (1,050–1,500 ms) may reflect the same ongoing ERP component is supported by their similar scalp distribution (see first and last voltage maps in Figure 6A).

To test this assumption statistically, we ran ANOVAs directly comparing the two time windows (i.e., including the additional factor TimeWindow). As expected, all significant effects involving the factor Condition were found to display the same scalp distribution in both time windows (100–300 ms and 1,050–1,500 ms, respectively), i.e., they did not interact with TimeWindow. At midline electrodes, we found a Condition × Electrode interaction [*F*_(3, 63)_ 3.49, *p* = 0.04], reflecting a frontal negativity [in Fz only, *F*_(1, 21)_ 5.59, *p* = 0.03], whereas lateral electrodes showed a main Condition effect [*F*_(1, 21)_ 4.96, *p* = 0.04]. In contrast, for the N400 between 400 and 500 ms, the ANOVA yielded significant Condition effects at midline and lateral electrodes, as well as a Condition × Laterality interaction at lateral electrodes (see Table 8). This interaction reflects a main Condition effect at medial electrodes. As a whole, this broadly distributed pattern along the midline strongly suggests the presence of a second (more posterior) negativity in addition to the ongoing frontal one. Lastly, in the P600 time window (800–1,050 ms), we observe a significant Condition × Hemisphere interaction along with higher-order interactions involving Condition, Hemisphere, Anteriority, and Laterality at lateral electrodes, and no effect at the midline. These interactions reflect a right-lateralized (and somewhat anterior) negativity, and a left-lateralized (somewhat posterior) positivity that largely cancel each other out at the midline (see third voltage map in Figure 6A).

**Table 8:** Global repeated measures ANOVAs for final consonant singular verbs (CONS) in neutral contexts (Trigger 4) at time-windows of interest.

			**(N400)**	**(P600)**	**Negativity**
		***df***	**400–500**	**800–1,050**	**1,050–1,500**
**NEUTRAL CONTEXTS, SINGULAR VERBS ONLY**					
Lateral	Condition	(1, 21)	6.88^*^	–	4.72^*^
	Condition × Laterality	(1, 21)	6.36^*^	–	–
	Medial: Condition	(1, 21)	9.30^**^	–	–
	Condition × Hemisphere	(1, 21)	–	5.68^*^	–
	Right Hem: Conditon	(1, 21)	–	6.70^*^	–
	Condition × Hem × Anteriority	(1, 21)	–	3.56^†^	–
	Condition × Hem × Laterality	(1, 21)	–	6.55^*^	–
	Right Hem: Condition	(1, 21)	–	6.67^*^	–
	Lateral: Condition	(1, 21)	–	5.85^*^	–
	Condition × Hem × Lat × Ant	(2, 42)	–	6.72^**^	–
	Left Hem: Con × Lat × Ant	(2, 42)	–	4.08^*^	–
	Left Hem, front: Con × Lat	(1, 21)	–	5.53^*^	**–**
Midline	Condition	(1, 21)	7.78^**^	–	–
	Condition × Electrode	(3, 36)	–	–	3.66^*^
	Fz: Condition	(1, 21)	–	–	5.54^*^

*Only significant results and trends are presented. Ant, Anteriority; Cent, Central; Con, Condition; Front, Frontal; Hem, Hemisphere; Lat, Laterality; Post, Posterior*.

† p < 0.10,

* p < 0.05, and

** *p < 0.01*.

For CONS *plural* verbs (depicted in Figure 6B, statistics in Table 9) we observe a number mismatch effect reflected by a more delayed N400 than in singular contrasts (650–800 ms after verb onset), followed by a frontal P3a-like positivity (800–900 ms) and a late posterior one (1,100–1,300 ms). We ran an ANOVA for plural CONS verbs in the later N400 time-window (650–800 ms). This yielded a significant Condition main effect at midline and a Condition × Anteriority interaction at lateral electrodes. Decomposition of this interaction revealed a main Condition effect at both central and posterior electrodes. An ANOVA in the 800–900 ms time-window yielded a Condition × Electrode interaction at midline, and a Condition × Anteriority interaction at lateral electrodes. These interactions reflect a significant frontal positivity (main effects of Condition at Fz), and a corresponding trend at anterior lateral electrodes. Finally, a main effect of Condition was found in the late P600 (1,100–1,300 ms) time-window, but only in posterior electrodes. No other main effects or interactions were found.

**Table 9:** Global repeated measures ANOVAs for final consonant (CONS) plural verbs in neutral contexts (Trigger 4) at time-windows of interest.

			**(N400)**	**(late N400)**	**(P600, frontal)**	**(P600, posterior)**
		***df***	**400–500**	**650–800**	**800–900**	**1,100–1,300**
Lateral	Condition	(1, 21)	–	–	–	–
	Condition × Anteriority	(2, 42)	–	4.36^*^	5.50^*^	–
	Anterior: Condition	(1, 21)	–	–	3.25^†^	–
	Central: Condition	(1, 21)	–	5.45^*^	–	–
	Posterior: Condition	(1, 21)	–	8.41^**^	–	10.05^**^
Midline	Condition	(1, 21)	–	5.47^*^	–	–
	Condition × Electrode	(3, 36)	–	2.88^†^	4.62^*^	–
	Fz: Condition	(1, 21)	–	–	4.50^*^	–

*Only significant results and trends are presented*.

† p < 0.10,

* p < 0.05, and

** *p < 0.01*.

##### Discussion for number mismatches on verbs

Whereas cross-modal lexico-semantic mismatches have been shown to elicit N400s in a number of previous studies, number mismatches between visual and auditory input have not been studied so far. Given that our paradigm used grammatical sentences it was unclear whether our number mismatches would elicit ERP profiles typical for morphosyntactic agreement violations, i.e., LAN/N400s and P600s. Number disambiguation in neutral contexts only became available on the verbs. Not unlike mismatch effects at sentence onset, ERPs at verb onset elicited biphasic (N400-P600) profiles in three out of four contrasts. As expected, component latency was influenced by the availability of disambiguating number information (earlier for verb-initial liaisons than for verb-final consonants, and earlier for shorter singular than for longer plural CONS verbs). In addition, two conditions (LIAS plural and CONS singular) displayed sustained anterior negativities, resulting in complex patterns of overlapping ERP components. In contrast, singular LIAIS mismatches did not display any systematic ERP effects at all.

For LIAIS verbs, we first discuss the lack of ERP components for the singular condition before turning to effects found in the plural.

##### Number mismatches on singular liaison verbs

The absence of ERP effects in the singular LIAIS condition corresponds to relatively poor behavioral performance in that particular condition, i.e., sentences with neutral contexts (e.g., “*For dessert, she likes…”* concurrently with an image illustrating two girls). The different ERP mismatch effects for singular vs. plural sentences with neutral contexts in LIAIS verbs may therefore reflect these difficulties. Note that we cannot explain these effects by appealing to differences between commission and omission, nor plural vs. singular forms (singular being the default), since CONS singular forms *did* elicit ERP components. Similarly rule strength or predictability would promote better perception of differences in liaison, as this process is obligatory in French, and also reliably occurs in determiner-noun contexts. We explore phonological salience, truth-value interpretations assigned to sentences, and sociolinguistic variability as explanations for these results.

Phonological salience (or perceptual salience) refers to the ease with which we can hear or perceive a given structure (Goldschneider and DeKeyser, 2005). Applied to our materials, we can expect that arriving at an accurate sentence interpretation is facilitated by overt phonological cues for number. We used an overt cue for number with LIAIS verbs, which in the plural is arguably more salient—due to the presence of a /z/—than in the singular without a /z/. It seems very unlikely that a participant—after hearing *elles aiment* [εl**z**εm]—would be willing to deny the cue's presence and assume she may have hallucinated, just because the picture only shows a single potential subject. However, if the same participant sees a picture with two girls and hears singular forms such as *elle aime* [εlεm], it seems possible to conclude to having misperceived liaison. Similar differences between the presence vs. absence of phonological (and visual) evidence have been found for prosodic boundaries and commas (leading to the “Boundary Deletion Hypothesis,” cf. Steinhauer and Friederici, 2001; Pauker et al., 2011). Phonological salience thus seems to provide a plausible explanation for the absence of ERP mismatch effects for singular sentences with neutral contexts. However, it does not account for all of our data, as singular CONS mismatches (which were also marked by a non-salient cue) did in fact elicit ERP responses.

Alternatively, the null result for LIAIS singular mismatches might be due to their enhanced acceptability, based on truth-values. Acceptability assigned to our sentences can be either logically or pragmatically motivated. For example, the sentence *Some triangles have three edges* is logically true, but under-informative and pragmatically odd. Similarly, when presented with an image of two girls eating chocolate mousse, describing the picture with “*She likes …”* is also logically true, but pragmatically odd. The ERP literature suggests that people differ in their bias toward logical vs. pragmatic processing (e.g., Barbet and Thierry, 2016). If some of our participants were biased toward logical processing, we would expect reduced or absent mismatch effects for neutral singular mismatching sentences. Crucially, however, even though one could argue that a lacking mismatch effect due to logical processing biases should be limited to singular sentences, there is no reason why it should be limited to sentences that are disambiguated by LIAIS verbs. That is, number mismatches disambiguated by CONS verbs would be subject to the same logic, but they did elicit clear ERP mismatch effects.

Yet another way of explaining the absence of ERPs for LIAIS singular mismatches comes from sociolinguistics. According to Prof. Julie Auger at Indiana University (personal communication), *elles* “she.plur” does not exist in informal Québec French, due to a process of neutralization (i.e., masculine and feminine plural pronoun clitics have become indistinguishable). Both are pronounced [i] before a consonant and [j] before a vowel (e.g., *les filles/les garcons y'aiment* “The girls/the boys, *they* like” are equally grammatical), although there is some variability between dialects. Two corpora from French monolingual speakers in Quebec City and bilingual speakers in Ottawa-Hull reveal few uses of *elles*, and omission or replacement of *elles* by *ils* “they.masc” in addition to /l/-deletion (i.e., /il/ or /ilz/ pronounced [i], [iz], or [j], but rarely [εl/z] or [ıl/z] the standard forms for plural) (Poplack and Walker, 1986; Bourget, 1987). The [j], being a semi-vowel, is licit before a vowel-onset verb and no additional liaison is necessary, and could in fact block liaison, since the verb onset is filled. Thus, perception of a subject-verb agreement error in liaison might be less systematic in singular conditions due to loss, or variability, of this grammatical feature, an interpretation that is coherent with our behavioral data where only these forms showed lower accuracy rates. We do not know of a psycholinguistic study that directly investigates liaison processing in Québec French, and so this interesting account remains somewhat speculative. While it appears to best explain our ERP null result for LIAIS singular mismatches (and is not applicable to CONS verbs), we should recall that participants still recognized the mismatches more than 85% of the time. We suggest that the absence of consistent ERP effects with LIAIS singular verbs reflects increased variability in processing strategies across participants, which may very well be influenced by sociolinguistic variability. As reflected by later sentence-wrap-up effects (see Supplementary Materials), in some cases error processing might also have been delayed.

##### Number mismatches on plural liaison verbs

The early-onset and sustained frontal negativity for plural mismatches resembles a classic morphosyntactic (dis-)agreement effect in auditory studies, possibly corresponding to more short-lived LAN-like effects in reading studies (Hasting and Kotz, 2008; Steinhauer and Drury, 2012). The extremely short onset latency of this effect, around 100 ms in our data, may be slightly overestimated due to possible co-articulation prior to the verb onset trigger (Trigger 4 in Figure 1) and the presence of the phoneme /z/ indexing a plural pronoun preceding it. As with Hasting and Kotz (2008), this is another illustration that morphosyntax-related processing difficulties that are clearly *not* driven by phrase structure violations can elicit this type of negativity (contra Friederici, 2002, 2011). Another similarity with Hasting and Kotz (2008), as well as many other auditory studies, is our finding of a complex pattern of overlapping ERP components (as discussed in Steinhauer and Drury, 2012). That is, sustained negativities are often superimposed by posterior P600 effects leading to a temporary mutual cancellation of components in at least certain electrodes. In our particular case, the negativity's scalp distribution in the early 100–300 ms time-window points to an even more complex pattern, as the P600 (500–800 ms) seems to be preceded by an additional, more posterior (N400-like) negativity from 100 to 300 ms that also overlaps with the frontal negativity. In our opinion, this is what explains the rather broad distribution of negativities in this time-window as reflected by statistical analyses, whereas the last portion of the “re-emerging” frontal negativity was limited to left-frontal electrode sites.

As with mismatch effects at sentence onset, the N400 effect may primarily indicate a lexical/phonological mismatch with what was predicted based on the picture. That is, participants saw a single person (e.g., one girl eating, thus predicting *elle* [εl], i.e., “she”) but heard sentences such as *Au dessert, elles aiment …* “For dessert, *they* like …”. Importantly, at least initially this mismatch is compatible with a number of interpretations. First, it is possible that the perceived mismatch included both the pronoun and the verb (*elles aiment* “they.fem.plur like” instead of *elle aime* “she.fem.sing likes”). This implies that the auditorily presented sentence as a whole was processed as a grammatical plural sentence, and the pronoun + verb as a whole mismatched across modalities. The first mismatching cue was provided by the pronoun at verb onset (liaison) and elicited an N400, as with NP contexts at sentence onset. The subsequent P600 was also triggered by the pronoun + verb and either reflected conflict monitoring and mismatch resolution or task-relevant categorization of a mismatching trial, or both. In this scenario, it is also possible that participants considered a *generic* interpretation. That is, “they (i.e., girls) like chocolate mousse” is an assertion that, in principle, could be illustrated with one single girl. As in English, French generic expressions are realized in the plural. However, for a generic (acceptable) interpretation we would predict a higher acceptability rate (which we did not find) and not expect a P600 (which we did find). Secondly, it is possible that the visual presentation of a single person activated a very strong expectation for a singular sentence. Knowing that incoming sentences were always supposed to describe the pictures, all spoken information up to phoneme /z/ at the liaison (including the entire context and most of the pronoun [εl]) was compatible with a singular interpretation, and it is conceivable that the longer the ambiguity lasted, the more this singular interpretation was strengthened. This expectation of a singular sentence may have led to two processing strategies that are both distinct from the first one discussed above: One is that only the pronoun, but not the verb, was processed as a plural form. Recall that liaison verbs were phonologically indistinguishable between singular and plural, i.e., *aime/nt* [εm]. So hearing *elles aiment'* [εlzεm] could have been interpreted as *elles* ^*^aime, “they likes,” a classical morphosyntactic agreement violation. In this scenario, the P600 would reflect some process of reanalysis toward a singular interpretation. The other possibility assumes that the initial expectation of a singular sentence was so strong that it led participants to temporarily mis-parse the incoming speech signal. Instead of interpreting /z/ as the pronoun plural marker (*elles* [εlz] + *aime(nt)* [εm]) they may have interpreted it as a verb-initial phoneme (i.e., *elle* [εl] + *zaime(nt)* [zεm]). This latter scenario is a possibility, as certain properties of French may have supported this. For instance, pronouns do not normally carry stress and are cliticized with the next content word to form one prosodic word where the content word carries word-final stress. Moreover, according to the “maximal onset principle” (Selkirk, 1981), the plural pronoun marker /z/ is syllabified into the verb's first syllable, as [εl.**zεm**] and not [εlz.**εm**] (bold font indicates stress). This is the same pattern one would expect for a singular utterance (i.e., *elle zaime*). Importantly, even though the verb *zaimer* does not exist in French, there are a number of French verbs that do start with /z/ (e.g., *zigonner* “to dally,” *zigouiller* “to kill,” *zigzaguer* “to zigzag,” *zézayer* “to lisp,” *zyeuter* “to observe intently,” *zébrer* “to decorate with stripes”). In other words, given the large number of different verbs used in our study (without any within-subject repetition), it is conceivable that in the LIAIS plural condition participants might have checked their lexicon for a verb that starts with /z/. We propose that ambiguity complexity in this particular condition may have elicited the additional sustained negativity, possibly reflecting evaluation of multiple options.

##### Number mismatches on singular consonant-final verbs

We will now turn to number mismatches on consonant-final verbs. The singular CONS mismatch condition with neutral contexts again elicited three components: a sustained anterior negativity (AN), an N400 and a small slightly left-lateralized P600. This pattern resembles that found in plural LIAIS mismatches with, however, a reduced P600. The later onset for the N400 as compared with LIAIS verbs can be straightforwardly explained by the later appearance of disambiguating information in the CONS condition's sound-streams. Interestingly the AN does not differ in distribution between early and late time-windows. According to Steinhauer and Drury (2012), this is one way of demonstrating that two negativities are likely early and late portions of the *same* (ongoing) ERP component. In the intervening time-windows, it is first superimposed by an N400 and then canceled out by a P600, which themselves may have overlapped and canceled each other out to some extent (explaining the absence of either effect between 500 and 800 ms). In contrast to both sentence onset and LIAIS verb conditions, here number ambiguities lasted until the verb-final consonant. That is, when participants saw a picture of two lions roaring and heard *En soirée il rugit* [ilʁyʒ**i**] *dans la savane* “In the evening he roars in the savannah,” only the lack of the verb-final consonant [s] (*rugissent* [ilʁyʒI**s**]) indicated a mismatch. Importantly, as the singular and plural pronouns *il* and *ils* are homophonous ([il]), we assume that the pronoun was initially processed as a plural (as suggested by the picture). Thus, one interpretation of what happened at the disambiguation point is that participants interpreted the auditory input as *ils* ^*^rugit, (“he.plur roar.sing”), which corresponds to a classical oral-language agreement violation. As before, the N400 would reflect a lexical-phonological mismatch, and the P600 would be associated with both categorization of this sentence as a mismatch and a potential attempt to revise its structure. Recall however, that (a) phonologically, the absence (omission) of a verb-final consonant is not very salient, and (b) participants were strongly biased toward a plural interpretation. Therefore, it is conceivable that participants were not entirely sure if the perceived mismatch was real or if they had simply missed an actually present consonant. Similar temporary confusions based on strong predictions are known from e.g., Itzhak et al. (2010) who demonstrated that listeners perceive a prosodic boundary in absence of any acoustic markers, if both lexical information and syntactic structure strongly predict it. Moreover, and only in the CONS singular condition, it is possible that participants initially parsed the subsequent preposition's word-initial consonant as a verb-final plural marker. In our example (*il(s) rugit* [ilʁyʒ**i**] *dans …*). Misinterpreting the /d/ of *dans* as a plural marker would result in [ilʁyʒi**d**], which could—in principle—be interpreted as a plural verb form (i.e., *ils rugident*). However, in the singular, the stem-final vowel is stressed due to the absence of a word-final coda (compare *ils rugissent* [ilʁyʒ**Is**]), and is a strong cue to word structure. At this point, participants would need to check this verb's stem forms in their mental lexicon and verify which one is legal in the plural. We believe that the complexity involved in this ambiguity is the reason why we find, once again, a sustained frontal negativity, resembling the LIAIS plural condition. As in previous conditions, we interpret the N400 as a reflection of an initial lexical-phonological mismatch, and the P600 as an attempt to resolve its structural consequences. The fact that the frontal negativity lasted beyond the P600 duration (as in LIAIS plurals and previous auditory agreement studies, e.g., Hasting and Kotz, 2008) suggests that the P600 does not always reflect the final stage of evaluation processes. One particularity of the CONS singular mismatch pattern was that the P600 itself did not reach statistical significance. Several previous studies have refrained from interpreting similar findings (e.g., Ye et al., 2006; Hasting and Kotz, 2008), but Steinhauer and Drury (2012) have argued that in the presence of ongoing negativities, the existence of a P600 can be inferred if this negativity is temporarily canceled out during the P600 time window (and at plausible electrode sites) and then re-emerges. We will come back to this point below.

##### Number mismatches on plural consonant-final verbs

Unlike singular CONS verbs, mismatches with plural CONS verbs elicited only a posterior N400 followed by a large P600, but no AN. As expected (see above), both components emerged slightly later than in the singular condition (due to the longer plural form duration). In many ways the plural condition resembles the singular one, however, the mismatching information is (a) phonologically salient and (b) an unambiguous plural verb marker. Thus, once plural information has been encountered, there can be no doubt that the verb is incompatible with an initial assumption of a singular pronoun (akin to a garden path sentence). In our example, the most likely lexical representation would be *En soirée il* ^*^rugissent [ilʁyʒI**s**]—a classical case of morphosyntactic number disagreement. In fact, we believe that—of all number mismatch conditions in our study—this condition is closest to a traditional oral-language agreement violation. As both the presence and the nature of this mismatch are extremely obvious, both the N400 and the P600 were found to be strong and consistent, while no AN reflecting effortful evaluation of a more ambiguous scenario was elicited.

#### Sentence-Final Negativities and Wrap-Up Effects

A subset of number-mismatch conditions (see Supplementary Materials), as well as the lexico-semantic condition, elicited a late posterior negativity at sentence end (1,700–2,000 ms), which we interpret as potential “sentence wrap-up” effects for both types of error. In contrast to positive waveforms that tend to occur in sentence-final positions of correct sentences, negativities are typically associated with preceding linguistic anomalies and may reflect additional processing load involved in reconsidering the anomaly and integrating the entire sentence (Osterhout and Mobley, 1995). A recent study from our lab on conceptual and logical semantic anomalies also showed that sentence final N400-like “wrap-up” effects are common, irrespective of the type of linguistic violation occurring in mid-sentence positions and of whether these elicited local N400s or P600s (Bokhari, 2015). Recently, Stowe et al. (2018) have raised the question of whether “sentence wrap-up effect” is an appropriate label for these negativities given the link to anomalies; these authors suspect that task requirements may also play a role in eliciting them. “Anomaly-related sentence-final negativity” may thus be a more neutral term to characterize these ERP effects.

## General Discussion

The present study used ERPs to investigate whether visual-auditory mismatches between a picture and a perfectly grammatical spoken sentence would elicit similar brain responses as typically seen for *within*-sentence linguistic anomalies. We included both cross-modal semantic mismatches, realized on verbs, and number mismatches (singular vs. plural) that occurred at different sentence positions using a range of linguistic number markers in spoken French (determiners, liaison, and verb-final consonants). Analyses also contrasted potential differences between singular and plural mismatches. Overall, our data demonstrate that cross-modal mismatches result in ERP profiles known from the literature for linguistic anomalies, and seem to distinguish between mismatches that can be described as purely conceptual-semantic and those that can be viewed as concerning grammar.

### N400s, P600s, and ANs—Evidence for Agreement Violations?

Returning to our initial research questions, our data have demonstrated that (a) cross-modal semantic mismatches realized on verbs elicit typical N400s and that (b) participants use the first available linguistic cues to detect number mismatches between a picture and a spoken sentence. Whether the ERP components found for cross-modal number mismatches are indistinguishable from those typically observed for “purely linguistic” within-sentence agreement violations, is less clear. On the one hand, all components we observed for number mismatches are within the range of ERP effects previously observed for morphosyntactic agreement violations. On the other hand, Molinaro et al. (2011a) reported that previous studies on number agreement violations have typically found LANs and P600s. While most of our negativities preceding the P600s did show a LAN-like frontal distributions, sometimes even with a left-lateralized prominence, statistical evidence usually pointed to a broadly distributed negativity compatible with an N400. Moreover, clearer evidence for left-anterior negativities (i.e., in LIAIS plural verbs) could be attributed to an early-onset sustained negativity at left frontal electrodes (e.g., F3). Overall, we believe our data are more compatible with an N400-P600 profile than with a LAN-P600 one. However, most previous ERP studies on number (dis-)agreement have focused on effects within NPs (determiner-adjective-noun) in the written modality. It is still controversial to what extent LANs (especially in reading studies) result from component overlap between N400s and P600s (e.g., Tanner and Van Hell, 2014). Nevertheless, our data do provide evidence showing that early-onset sustained negativities in mismatch studies can show a clear left-anterior distribution that cannot be explained by component overlap. Since LANs in reading studies tend to have latencies and durations comparable to N400s (i.e., 300–500 ms), we are increasingly less convinced that sustained (left-)anterior negativities in auditory studies (e.g., Brink and Hagoort, 2004; Hasting and Kotz, 2008) are analogous to LAN components in reading studies. For our current data, we suggest that sustained negativities may index a continued evaluation of more complex cases of ambiguity resolution. The N400s we found virtually in all number mismatch conditions are rather difficult to interpret with confidence, as various accounts would predict N400-like components, including for standard morphosyntactic violations involving predictable inflectional morphemes (e.g., Tanner, 2015; BSS2019), truth-value related approaches (Bokhari, 2015), and phonological mismatch accounts (Connolly and Phillips, 1994). Molinaro et al. (2011a) have argued that phonotactics involved in agreement processes might demote grammatical processing (reflected by LANs) toward a lexical one (reflected by N400s). Our CONS verbs had a variety of final consonants (9 different consonants over our 60 verbs). These consonant changes do not follow systematic morphological rules. They are sometimes described as consonant deletion rules from the plural to the singular (Paradis and El Fenne, 1995). However, since singular forms are the default (and are acquired first), Royle (2011) argues against this approach and proposes rather that consonant-alternating forms in French are lexicalized (her research focused on adjectives, but the same logic can also be applied to verbs). This could promote use of lexical rather than grammatical processing when checking agreement, and thus explain N400 effects observed in plural conditions.

## Conclusion

With the aim of testing whether cross-modal mismatches between pictures and *grammatical* sentences would elicit similar ERP components to those in the literature on linguistic anomalies, we developed an experiment with auditory-visual sentence-picture matching paradigms and an acceptability judgment task in French. We investigated neurocognitive mechanisms underlying lexico-conceptual semantics and grammatical number processing. This is the first study to test three different linguistic cues for number mismatches at different sentence positions. Our results demonstrated that native French speakers reliably exhibit N400 components in response to cross-modal verb-action mismatches, comparable to previous effects found for noun-object mismatches. Auditory-visual number mismatches usually elicited a biphasic N400-P600 (in some cases superimposing a sustained AN), and our context manipulation demonstrated that participants use the first available sentence cue to disambiguate structures. ERP effects at sentence onset and on the verb suggest that participants immediately tracked mismatches between modalities as soon as conflicting information became available, and that these mismatches were processed in a way that is not fundamentally different from purely linguistic within-sentence agreement violations.

Our paradigm is exciting for a number of reasons, one being that we used grammatical sentences to induce “agreement error” processing, and elicited well-known ERP components. This approach has the advantage of being more ecologically valid than error-based paradigms, as it resembles more closely the mostly error-free speech we are exposed to daily. Having developed this experiment for younger populations, we are confident that our approach will reveal, in children, what types of information are being used at which point in the speech stream to disambiguate information. This type of paradigm also has potential for the study of developmental language disorder as well as second-language learning, as is the visual-world paradigm used in eye-tracking studies (e.g., Hopp and Lemmerth, 2018).

We can anticipate future directions of inquiry from this initial study of verb-based visual-auditory mismatches. As we have seen, not all incongruent number mismatch conditions elicited strong P600s despite the fact that we used a judgment task, which promotes this component. The N400 component seemed to be a more reliable reflection of our mismatch errors. This might in part be due to the fact that we did not use ungrammatical sentences as input, reducing error-detection based strategies that could have been used in most studies that find the LAN or the P600. Our robust N400s instead of LANs (or ANs), and less robust P600s for mismatches, might be the result of our sentences' *grammatical* status.

As we have appealed to sociolinguistics to explain some of our results, it appears interesting to pursue sociolinguistic studies using ERPs. This combination of domains has rarely been explored and we can identify straightforward implementations, as in second language acquisition research, to study variability in grammars within geographically constrained but linguistically diverse speakers of the same language. Paying attention to how a speaker implements a particular linguistic rule has strong potential to help us better understand the neurocognitive underpinnings of within-group variability in language processing.

In conclusion, our study provides a significant contribution to the field of cognitive neuroscience of language by providing high-quality evidence regarding the generalizability of ERP profiles across modalities and languages. This study extends lexico-semantic mismatches to the domain of verbs, provides insight into context effects and early detection of mismatches, establishes ERP patterns for different types of morpho-phonological and morpho-syntactic cues for number mismatch processing, and demonstrates that even grammatical sentences can elicit ERP patterns associated with “error” processing.

## Ethics Statement

This study was carried out in accordance with the recommendations of McGill's Institutional Review Board (IRB) and University of Montreal's Comité d'éthique à la recherche en Santé of the Faculty of Medicine, with written informed consent from all subjects. All subjects gave written informed consent in accordance with the Declaration of Helsinki. The protocol was approved by the McGill's Institutional Review Board (IRB) and University of Montreal's Comité d'éthique à la recherche en Santé of the Faculty of Medicine.

## Author Contributions

ÉC and LM collected the data, performed the analyses and the statistics, and should equally be viewed as first authors. LM wrote the first draft of the introduction, the methods section, and certain sections of the results as a part of her master's thesis. ÉC, PR, and KS wrote and edited the final version of the paper. KS and PR developed the general idea for the study and oversaw all stages of data analysis. PR, ÉC, and KS developed the theoretical background and issues addressed in the paper, and wrote the results and discussion of the manuscript. All authors designed the experiment and contributed to manuscript revision, read, and approved the submitted version.

### Conflict of Interest Statement

The authors declare that the research was conducted in the absence of any commercial or financial relationships that could be construed as a potential conflict of interest.

## Footnotes

^1^Note that in oral Quebec French, a subject with an overt NP “The girl” followed by a pronoun “she” is grammatical (some say the pronoun is obligatory) contrary to written French.

^2^http://www.rstudio.com/

^3^http://CRAN.R-project.org/package=ez
