# Corrigendum: Eliciting ERP Components for Morphosyntactic Agreement Mismatches in Perfectly Grammatical Sentences

**DOI:** 10.3389/fpsyg.2020.00860

**Published:** 2020-05-05

**Authors:** Émilie Courteau, Lisa Martignetti, Phaedra Royle, Karsten Steinhauer

**Affiliations:** ^1^Faculty of Medicine, School of Speech Language Pathology and Audiology, University of Montreal, Montreal, QC, Canada; ^2^Centre for Research on Brain, Language and Music (CRBLM), Montreal, QC, Canada; ^3^Faculty of Medicine, School of Communication Sciences and Disorders, McGill University, Montreal, QC, Canada

**Keywords:** subject-verb number agreement, event-related brain potentials (ERPs), auditory-visual sentence-picture matching paradigm, cross-modal number mismatches, French language, online grammaticality judgment, N400 and P600, sustained frontal negativity

In the original article, there was a mistake in Figure 4 as published. Instead of correctly describing effects in singular (A) and plural (B) NPs, as in the figure caption, the legend incorrectly describes NP contexts in (A) and neutral contexts in (B). The corrected Figure 4 appears below.

**Figure 4:**
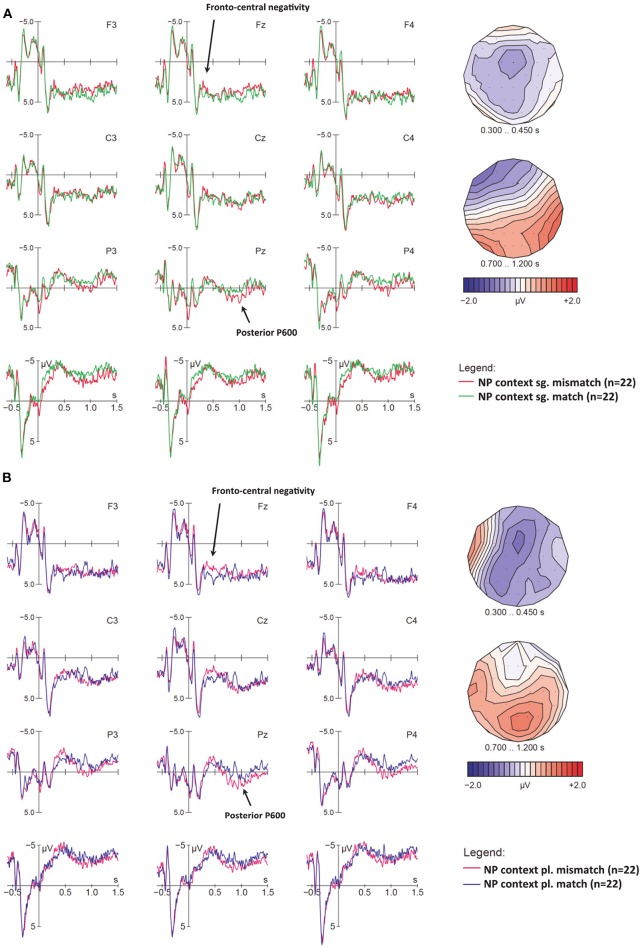
Early effects of cross-modal number mismatches in NP contexts, for **(A)** singular and **(B)** plural NPs at sentence onset. ERPs are time-locked to the onset of the determiner (vertical bar) with a baseline of −600 to 0 ms; voltage maps illustrate the difference waves of relevant effects. **(A)** Singular mismatches (red) elicited a small fronto-central negativity in the N400 time-window relative to singular matches (green), as well as a parietal P600. **(B)** Plural mismatches (magenta) elicited a larger N400 as well as a parietal P600, as compared to plural matches (blue). Voltage maps of these effects (mismatch minus control) show that singular and plural mismatches elicited quite similar components.

The authors apologize for this error and state that this does not change the scientific conclusions of the article in any way. The original article has been updated.

