# Supplementary material for: Eliciting ERP Components for Morphosyntactic Agreement Mismatches in Perfectly Grammatical Sentences
Source: Front Psychol. 2019 Jun 25;10:1152. doi: 10.3389/fpsyg.2019.01152 (PMC6613437; doi:10.3389/fpsyg.2019.01152)
Supplement: Supplementary file 1 [file Data_Sheet_1.docx]

Supplementary Materials

Eliciting ERP Components for Morphosyntactic Agreement Mismatches in Perfectly Grammatical Sentences

Émilie Courteau, Lisa Martignetti, Phaedra Royle, Karsten Steinhauer*

*** Correspondence:** Corresponding Author: Karsten.Steinhauer@mcgill.ca

# Supplementary Data

Sentence structures: 180 French verbs acquired before the age of 8 years were selected from the Manulex database (Lété, Sprenger-Charolles, & Colé, 2004). Both Manulex and Lexique (New, Pallier, Ferrand, & Matos, 2001) were consulted to provide oral language frequency norms for selected items, since the experiment will ultimately be used with children. Of the 180 verbs selected, one third were intransitive and were followed by a prepositional phrase (PP) (e.g., *Le lion, il rugit dans la savanne*, ‘The lion, he roars in the savannah’), one third were transitive (e.g., *La fille, elle aime la mousse au chocolat*, ‘The girl, she likes chocolate mousse’) and one third were ditransitive (e.g., *Le dompteur, il lance une balle à l'otarie*, ‘The tamer, he throws a ball to the sea lion’). Transitive and intransitive verbs were followed by an NP or a PP (or both). Note that to maintain equal sentence length across verb argument-structure types, certain transitive verbs were followed only by an NP. These account for 19% (15/77) of transitive verbs or 8% (15/180) of the verbs used. Verbs were inserted into sentences containing singular or plural third person subject pronouns (*he/she/they*), and a sentence continuation phrase consisting of a direct object NP, or a prepositional phrase (e.g., … *in the public pool*), to postpone wrap-up effects in the ERPs (Hagoort, 2003). Context phrases (neutral – e.g., *Each week* – and subject NP – e.g., *The lion*) were also created. We ensured that there were no additional instances of liaison in sentences or carrying phrases, such that only sentences in the liaison verb sub-condition contained them. We also ensured that all nouns, adverbs, prepositions and adjectives included in sentences and carrying phrases were age appropriate for children, as per Manulex (Lété et al., 2004). Further, subject grammatical gender (feminine or masculine), as well as syllable length of context phrases and of full sentences were balanced across the three verb categories.

Stimulus recording: Auditory recording, normalizing, and splicing was performed by trained research assistants with a background in speech editing. Auditory stimuli were recorded in a sound-shielded audiology booth using a Sony DAT recorder (PCM-M1 recorder, 1997). All sentences were spoken by a native French Canadian actor who was trained to pronounce words with clear but natural articulation, while maintaining constant and natural prosody and intonation. Sentences were spoken with natural within- and between-word co-articulation, however this was avoided at splicing points (e.g., between the last word in context phrases and the first word in the following sentences). The actor was also instructed to maintain a constant vocal intensity, intonation, and speech rate, throughout the recording. Voice-volume monitoring (+ or – 5 dB) was performed during recording. All conditions within a given block of stimuli were recorded together. All of this allowed us to create very natural-sounding auditory stimuli, while reducing post-recording manipulations.

Sound processing: Before cross-splicing took place, context phrase and sentence files were processed and normalised in five steps using Audacity® (Audacity Team, 2018) and Praat (Boersma & Weenik, 2018) software: (1) we reduced noise, such as microphone feedback and other extraneous sounds that were present in the recordings, by applying Audacity’s built-in Noise Reduction function to all files. During this process, care was taken to use the lowest level of manipulation possible in an effort to restrict distortion to the resulting audio files. For example, the sensitivity (which controls how much of the audio is considered noise) and frequency smoothing (which spreads the noise reduction process to the specified number of neighbouring frequency bands) options were always maintained at lowest possible levels; (2) as noise reduction created new instances of ‘silence’ at the start and end of audio files, such silences were trimmed using the Audacity *Trim Silence* plug-in. A threshold of -35 dB was selected, as this level was deemed most appropriate in removing silent portions, while sparing phonemes with lower amplitudes such as voiceless stops, during tests performed on a subset of files; (3) using a Praat script (created in-house by the second author), sentence onsets and offsets were then trimmed at zero-crossings –the point where the waveform crosses the zero-level axis– in order to avoid discontinuities in the sound wave which can be perceived as clicks or pops; (4) finally, we listened to each file to assess the naturalness of speech rate. In cases where speech rate was perceived to be too fast or slow in comparison to the majority of audio files, or where there was a discontinuity between context phrases and associated sentences, speech rate was adjusted by using Audacity’s built-in *Change Tempo* function. The lowest level of manipulation necessary to create natural sounding and consistent speech was used (maximum 15% slower or faster); (5) lastly, we added silent portions to the onsets and offsets of all audio files using Audacity’s *Trim/Extend* plugin, such that all final audio files (once context phrases and sentences were cross-spliced) contained 0.035 seconds of onset silence, 0.05 seconds of offset silence, and 0.1 seconds of silence between the context phrase and the rest of the sentence (these values were perceived to be most natural-sounding when tests were performed on a subset of audio files). Once context phrase and sentence processing was complete, they were cross-spliced to create audio files for different experimental conditions, as outlined in the next sections. Cross-splicing was carried out in Praat by means of a script (created in-house by the first author) which concatenated all context-phrase and sentence files based on conditions outlined above. Lastly, the amplitude of the resulting audio files was normalised to a level of 70 dB SPL using Praat.

Developing stimuli for the semantic condition: Items for the matched condition included 60 different verbs and were created in three steps. First, a native French Canadian actor spoke each congruent item, consisting of a context plus sentence combination. We then cross-spliced the neutral and subject NP contexts, as depicted in Table 1 of our article, examples (1a) - (1b), and (2a) - (2b). Each item pair was associated with one image depicting the action described in the audio files. Items for the mismatched conditions were created by cross-splicing the contexts from one item pair with the sentences from another, as depicted in examples (2a) - (2b), resulting in 120 mismatched item pairs. In the end, 60 item quadruplets were created for a total of 240 items (120 matched and 120 mismatched). In order to create semantic mismatches, each item quadruplet was associated to the same image, such that both matched and mismatched item versions were displayed with the same image.

Developing stimuli for the number mismatch condition: First, we recorded a subset of congruent items including 120 different verbs, namely a singular subject NP context and singular sentence combination (as in Table 2 of our article, Item 1b), a neutral context and singular sentence combination (Table 2, Item 1a), and a plural subject NP context and plural sentence combination (as in Table 2, Item 2b). This resulted in 120 sentence triplets (120 with singular subject NP contexts and singular sentences, 120 with plural subject NP contexts and plural sentences, and 120 with neutral contexts and singular sentences), for a total of 360 spoken items. We then cross-spliced as in the example provided in Table 2: each singular sentence was spliced with both a singular subject NP and neutral context (as in 1a and 1b), and each plural sentence was spliced with a plural subject NP context and the same neutral context that was used for the singular (as in 2a and 2b). This created item quadruplets, whereby the two singular versions differed in context only, the two plural versions also differed in context only, and the same neutral context was paired with both singular and plural sentences. This resulted in 120 item quadruplets, or 480 items. Each singular and plural item pair was associated with a corresponding image (i.e., singular items were paired with an image depicting one agent, and plural items were paired with an image depicting two agents). Number mismatches were created by presenting the same grammatical audio files, but swapping the images, such that audio files describing singular subjects were presented with images depicting plural subjects (Table 2, items 1c-d and 2c-d), and vice versa. In total, 960 audio file and image combinations were created (480 congruent and 480 incongruent).

List creation: Four different presentation orders (lists) were created. In each list, stimuli were evenly distributed across 15 blocks of 20 items each. The following constraints were met: (1) each block contained two items in each of the eight morphosyntactic sub-conditions as well as one sentence in each of the four semantic sub-conditions; (2) there was no consecutive repetition of the same sub-condition; (3) match and mismatch conditions were evenly distributed across each block; (4) in order to minimize strategic processing effects, pseudorandomization within blocks also prevented (a) consecutive presentation of items with the same agent, (b) consecutive presentation of more than three items from congruent or incongruent conditions, (c) consecutive presentation of more than three singular or plural items, (d) clusters of particularly long or short sentences. In order to further rule out any sequence effects, four additional mirror versions of each list were created by reversing both the block order and the sentence order within each block. Thus, a total of eight experimental lists were created and evenly assigned across male and female participants.

#### Sentence wrap-up effects for number mismatch conditions

As in the semantic mismatch condition, number mismatches also elicited posterior negativities at sentence-final positions that are compatible with a wrap-up N400 effect (see Supplementary Figures 1 and 2). A global ANOVA on the 1700–2000 ms time-window comparing match and mismatch verbs (both CONS and LIAIS) yielded significant Condition×Anteriority (*F*(2, 42) = 4.19, *p* = 0.04), and Condition×Laterality (*F*(1, 21) = 6.36, *p* = 0.02) interactions in the lateral electrodes. These interactions reflect the negativity’s posterior and medial distribution. While no significant interaction with Context was found, separate plots for the two contexts suggested that the wrap-up effect was more prominent for NP contexts. In fact, separate analyses revealed no clear wrap-up N400 effect for neutral contexts. In contrast, a comparison of match and mismatch verbs within NP contexts again reveals significant interactions of Condition×Anteriority (*F*(2, 42) = 5.56, *p* = 0.02), and Condition×Laterality (*F*(1, 21) = 4.70, *p* = 0.04), in lateral electrodes. These reflect the fact that a main effect of Condition is observed in posterior electrodes (*F*(1, 21) = 4.60, *p* = 0.04).

**
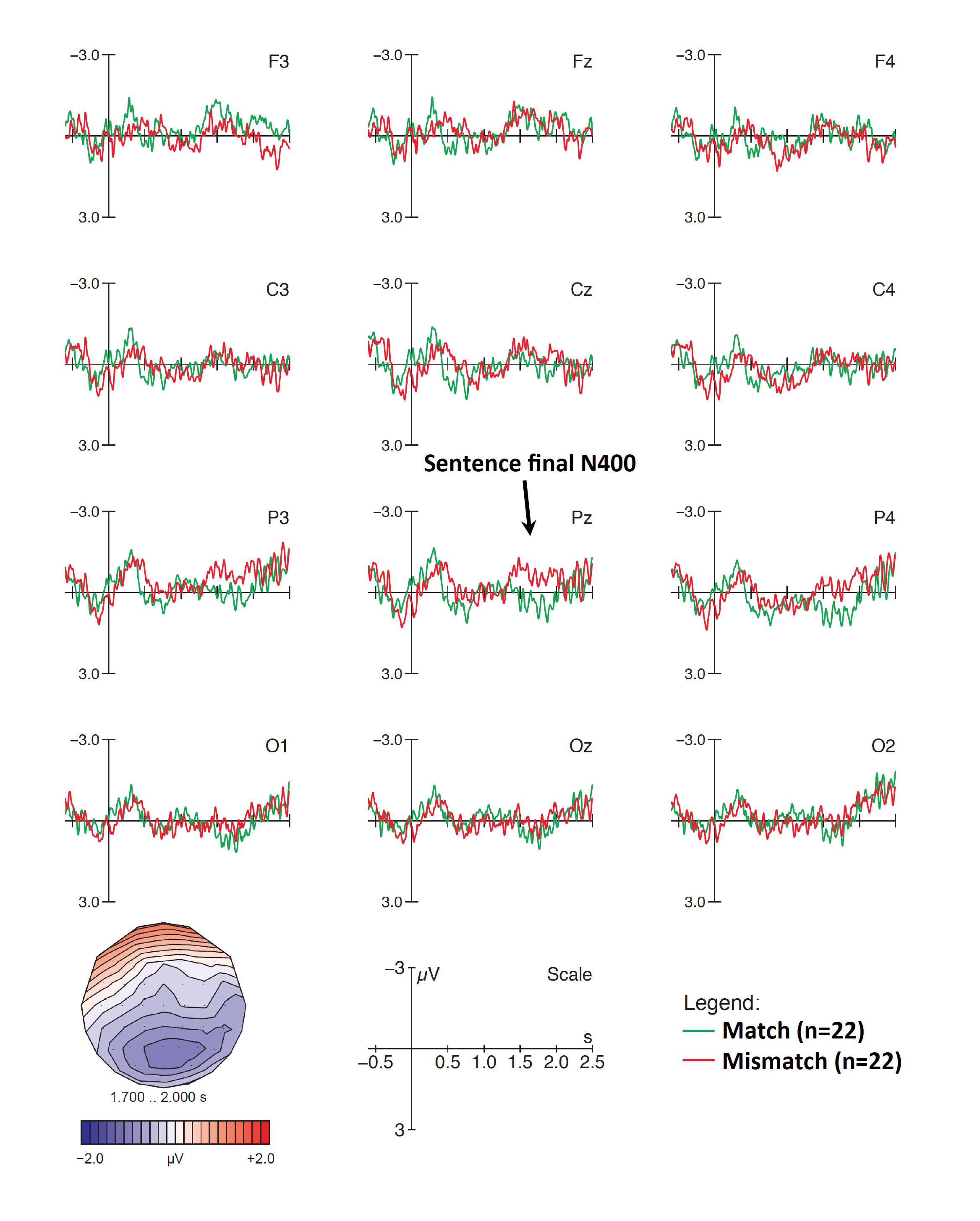
Supplementary Figure 1: Sentence wrap up effects in number mismatch conditions for NP contexts.** Late effects for cross-modal number mismatches in NP contexts measured from the onset of the verb. Displayed are grand-average ERPs at midline and medial electrodes for all participants, time-locked to the onset of the verb (vertical bar) with a baseline of -600 to 0 ms. Mismatching NP context conditions (red) elicited a late right-lateralized posterior negativity in the 1700–2000 time-window relative to matching NP contexts (green).


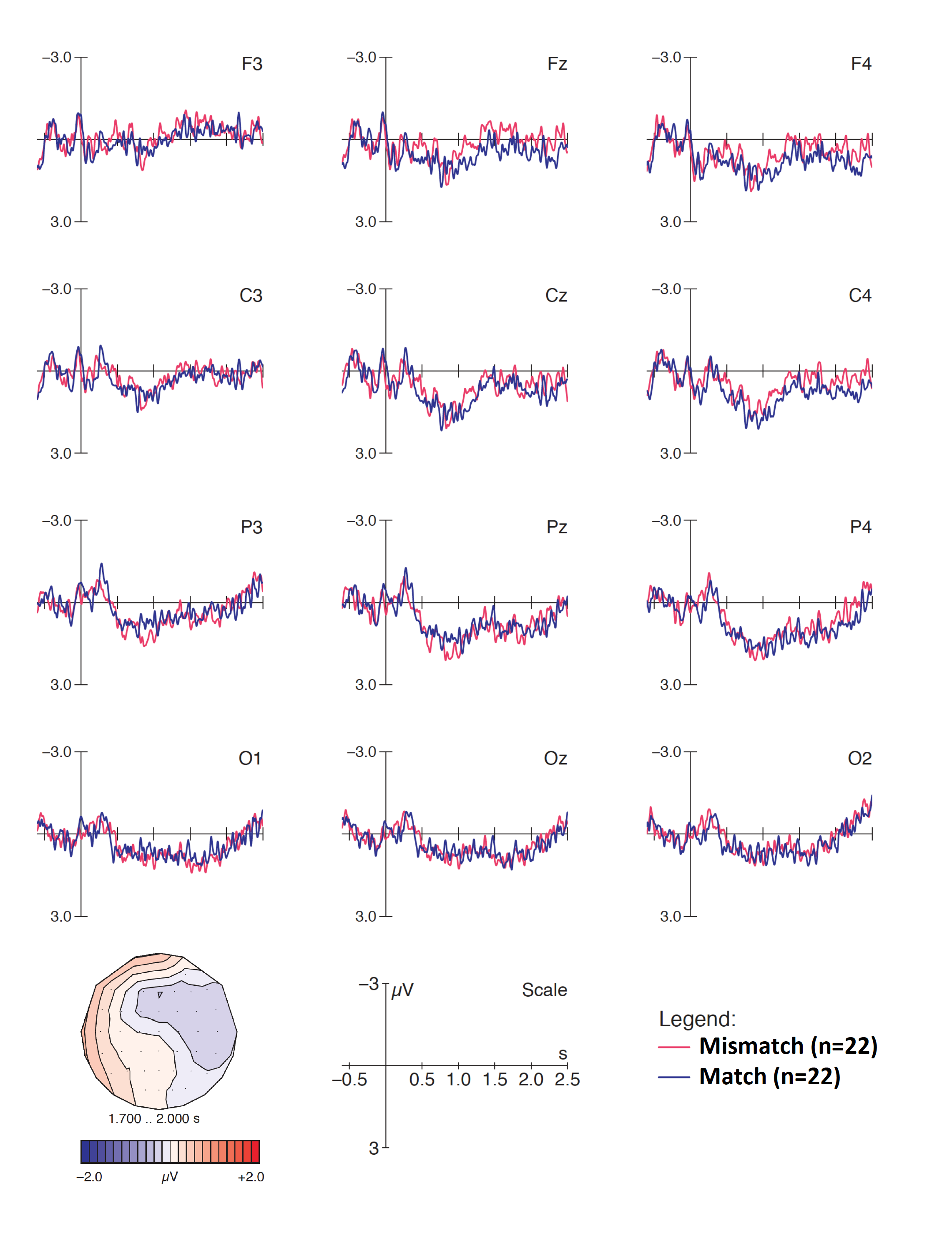


**Supplementary Figure 2: Sentence wrap up effects in number mismatch conditions for neutral contexts.** Late effects for cross-modal number mismatches in neutral contexts measured from the onset of the verb. Displayed are grand-average ERPs at midline and medial electrodes for all participants, time-locked to the onset of the verb (vertical bar) with a baseline of -600 to 0 ms. Mismatching neutral context conditions (magenta) elicited no negativity in the 1700–2000 time-window relative to matching neutral contexts (blue).

References :

Audacity Team (2017). Audacity(R): Free Audio Editor and Recorder [Computer application]. Version 2.2.1 retrieved December 20th 2017 from https://audacityteam.org/

Boersma, P., & W., David (2018). Praat: doing phonetics by computer [Computer program]. Version 6.0.40, retrieved September 2018 from http://www.praat.org/.

Hagoort, P. (2003). Interplay between syntax and semantics during sentence comprehension: ERP effects of combining syntactic and semantic violations. *Journal of Cognitive Neuroscience*, *15*(6), 883-899. doi:10.1162/089892903322370807

Lété, B., Sprenger-Charolles, L., & Colé, P. (2004). MANULEX: A grade-level lexical database from French elementary school readers. *Behavior Research Methods, Instruments, & Computers, 36*(1), 156–166. doi:10.3758/bf03195560

New, B., Pallier, C., Ferrand, L., & Matos, R. (2001). Une base de données lexicales du français contemporain sur internet : LEXIQUE. *L'Année Psychologique, 101*, 447-462. doi: 10.3406/psy.2001.1341
